# Investigation of COVID-19 Vaccine Information Websites across Europe and Asia Using Automated Accessibility Protocols

**DOI:** 10.3390/ijerph19052867

**Published:** 2022-03-01

**Authors:** Jinat Ara, Cecilia Sik-Lanyi

**Affiliations:** Department of Electrical Engineering and Information Systems, University of Pannonia, Egyetem u. 10, 8200 Veszprem, Hungary; lanyi@almos.uni-pannon.hu

**Keywords:** web accessibility, accessibility guidelines, automated tools, Mauve++, Nibbler, WAVE, WEB accessibility, information technology, COVID-19

## Abstract

Websites content accessibility guidelines (WCAG) ensure that websites should be perceivable, understandable, navigable, and interactive. During the SARS-CoV-2 pandemic, the importance of accessible websites and online content grew throughout the world. Therefore, in this study, we examined COVID-19-related official government websites. This research covered 21 government websites, with 13 websites from European countries and 8 websites from Asian countries, to evaluate their accessibility following WCAG 2.0 and WCAG 2.1 guidelines. The overall goal of this study was to identify the frequent accessibility problems that might help the website owners to identify the shortcomings of their websites. The target websites were evaluated in two steps: in step-1, evaluation was performed through four automatic web accessibility testing tools such as Mauve++, Nibbler, WAVE, and WEB accessibility tools; in step-2, evaluation went through human observation, such as system usability testing and expert testing. The automatic evaluation results showed that few of the websites were accessible; a significant number of websites were not accessible for people with disabilities. In addition, system usability testing found some complexity in website organization, short explanations, and outdated information. The expert testing suggested improving the color of the websites, organization of links, buttons, and font size. This study might be helpful for associated authorities to improve the quality of the websites in the future.

## 1. Introduction

The world wide web (WWW) was designed in 1989 to make information accessible to people [1]. In recent days, a large amount of data has grown faster on the cloud, and people are engaging online rapidly [2,3,4]. Therefore, the accessibility of online information is a crucial aspect for technologists and researchers. Nowadays, several past studies revealed that a large amount of online content such as websites are not accessible for people with disabilities [3,5]. Generally, the website is a digital platform for sharing a variety of information (healthcare, education, e-commerce, etc.) with the people. However, sometimes, these digital resources are not properly organized (such as the presence of broken links, out-of-date content, dilated images, etc.). From the perspective of digital services, the digital resources should be organized to make them accessible and barrier free to the people with disabilities [6]. Thus, to ensure accessible digital services, it is important to ensure that people with disabilities are considered. Their needs should be incorporated in the design and implementation of the web application.

Generally, web accessibility suggests that a website should have an advanced design and the latest technology for development so that people with disabilities can perceive, understand, navigate, and interact with the web more efficiently [7]. The W3C Web Accessibility Initiative (WAI) is an organization that introduces accessibility guidelines known as web content accessibility guidelines (WCAG) [8,9]. It provides WCAG 1.0, WCAG 2.0, WCAG 2.1, WCAG 2.2 and WCAG 3.0 standards for three conformance levels A, AA, and AAA. The WCAG aims to ensure accessible websites or content for people with disabilities [10,11,12,13,14].

**Figure 1:**
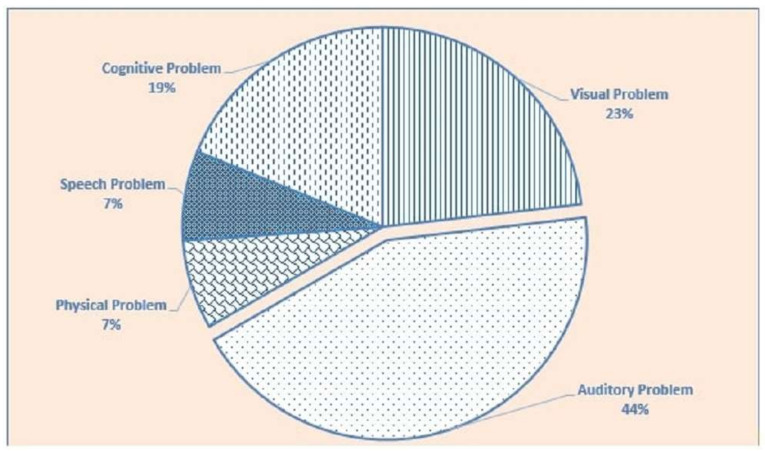
Worldwide ratio of people with several disability.

According to the World Bank statistics, one billion people (15% of the world population) across the world have some disabilities such as visual problems (vision impairment or vision loss), auditory problems (hearing disability), physical disabilities (disorder in a body part), speech problems (disorder speech production), cognitive or neurological problems (disorder in cognitive functions) and others [15]. According to the report of the World Health Organization (WHO), among the whole population of the world, 3.2% of people have a visual impairment, 6% of people have auditory or hearing difficulties, 2.6% of people have a neurological difficulty, and 1% of people have physical disabilities or require a wheelchair [16]. Based on the statistics of WHO, Figure 1 shows that, for every 100 people, 44 people have auditory, 23 people have visual, 19 people have cognitive, and 7 people have speech and physical issues throughout the world. According to EU statistics, in Europe, one in six people have some disabilities who are actively involved online to search for information [17]. The United Nations reported that, in the Asian region, more than 10% of the people who have disabilities are actively involved in internet platforms for information [18]. 

In 2019, the whole world experienced the SARS-CoV-2 virus. It was first detected in Wuhan, China, and spread rapidly throughout the world [19,20]. To control this virus, governments of different countries have started a vaccination program from 14 December 2020. To share the updated COVID-19 vaccine information and to facilitate the vaccination process, the governments of several countries have created their websites. These web-based portals aim to share COVID-19 vaccine information with the people. It helps people understand the vaccines and the importance of vaccination to fight against COVID-19. These government websites are considered to be official sources of COVID-19 vaccine information. Generally, all governments have individual regulations and publish all the information on their official websites, including the prioritized vaccine for senior people/government workers/social workers and others. In some countries, they have age limitations; some require vaccine registration or take online appointments, etc. Therefore, COVID-19 vaccine information seekers are relying on these official portals. In the context of digital accessibility and disability, statistics show that a significant number of people with a disability have accessed the online portal frequently [16]. Thus, accessibility is more important for COVID-19 vaccine information portals because restrictions in COVID-19 vaccine information access might reduce the social support of people with disabilities. Considering these issues, it is important to understand the accessibility of the official government-operated COVID-19 vaccine information websites and to implement inclusive design and development practices to make digital COVID-19 vaccine information portals barrier-free for people with special needs. These deliberations emphasized investigating and understanding the accessibility of official governmentCOVID-19 vaccine information websites to make the COVID-19 vaccine information available to all.

Few researchers from different countries (US, UK) have performed research on the accessibility of COVID-19 vaccination/registration portals [21]. They suggested that a significant number of official and private portals have accessibility issues. The majority of the countries have no strict rules or monitoring system to control these inaccessible websites that create massive barriers for the people who have disabilities. Therefore, researchers emphasized conducting comprehensive research on government and private COVID-19 vaccine information websites to validate their accessibility. Several organizations have showed their impressive effort and created their own websites for sharing information about the COVID-19 vaccine. This research is limited to the investigation of government-operated official COVID-19 vaccine information websites, as they tend to be more reliable, effective and accepted by the community.

By the last few years, researchers developed several online web accessibility testing protocols or tools that help the online content authority to understand and assess the accessibility of their online platform [22,23]. Among numerous web accessibility testing protocols: AChecker, Mauve++, The Accessibility Management Platform (AMP), TAW, WAVE, HTML Validator, and Nibble are more popular and have been used by previous researchers [19]. All the developed accessibility evaluation tools ensure fast and accurate accessibility scores, are cost-effective, and produce reliable evaluation results. Thus, identifying the appropriate and effective tools is challenging and a critical aspect. Therefore, our prime objective of this research is to analyze government COVID-19 vaccine information websites using multiple web accessibility testing protocols. In this research, we employed multiple web accessibility tools, as it is easier to compare and validate the result more effectively. Therefore, this study considers four popular open-source web accessibility testing tools, Mauve++, Nibbler, WAVE, and Web, to evaluate 21 official governmentCOVID-19 vaccine information websites from different parts of Europe and Asia. 

Furthermore, we validated the targeted websites through questionnaire-based human observations, including system usability testing and expert testing. Based on our findings, we offer some suggestions for the authorities involved with the COVID-19 information websites and web developers to improve the accessibility of their websites. This research might help to improve the website’s accessibility for people with disabilities and make the COVID-19 vaccine information freely accessible without any barriers. Throughout the evaluation, we tried to answer four research questions:

**Research** **Question** **1** **(RQ1).** *Is there any difference in the countries’ websites accessibility between the two continents in their average number of errors? What about the error if we focus on the website of each country?*

**Hypothesis** **1** **(H1).** *There is no difference between European and Asian countries’ websites according to their average number of errors. There is a difference if we focus on the website of each country.*

**Research** **Question** **2** **(RQ2).** *Is there any difference between European and Asian countries websites based on human judgment results?*

**Hypothesis** **2** **(H2).** *There is no significant difference between websites from the Europe and Asia considering questionnaire based human judgment results.*

**Research** **Question** **3** **(RQ3).** *Is there any difference in the result of automatic testing and human judgment result?*

**Hypothesis** **3** **(H3).** *There is a significant difference in the result of automatic testing and questionnaire-based human judgment.*

**Research** **Question** **4** **(RQ4).** *Which are the most frequently violated principles based on the error number of Mauve, Nibbler, WAVE, and WEB accessibility?*

**Hypothesis** **4** **(H4).** *The first principle is the most frequent violation principle considering four automatic testing tools.*

The remaining part of the paper is organized as follows: in Section 2, we describe several past studies associated with website accessibility considering methods, tools, etc. Section 3 represents the accessibility evaluation framework in detail: website accumulation process, web accessibility tools, and their functional structure. Section 4 present the test results with evaluation metrics, conformance level, principles, and potential errors. In Section 5, we will discuss our evaluation result and answer our research questions. In Section 6 and Section 7, we will discuss our major findings and provide some suggestions for future improvements, respectively. Finally, in Section 8, we will discuss the conclusion and our future work.

## 2. Background Study

Generally, there are several techniques or methods to evaluate web accessibility. Four web accessibility evaluation techniques are more popular and effective: automatic evaluation tools [24], manual evaluation or expert evaluation [25], user evaluation (those who have disabilities) [26], and hybrid evaluation (both automatic and manual judgment). The automatic evaluation technique allows the tester to evaluate the website’s accessibility by running the evaluation tools into the browser. It also refers to software which determine the quality of the websites based on predefined web accessibility standard guidelines [27]. Manual evaluation is performed using human experts to evaluate websites accessibility. Usually, manual evaluation or observation helps to identify the limitation of the automated accessibility testing tools. Sometimes, automated tools cannot determine all the accessibility issues according to the accessibility guidelines and, thus, require human judgments. Website accessibility testing with users having disabilities refers to user testing or testing assisted by aid users. In this aspect, users with disabilities can interconnect with website testing directly. The users with disabilities are invited and asked to evaluate the web pages at their discretion. Sometimes this testing procedure is more reliable than other manual or automatic testing processes. Furthermore, hybrid website accessibility testing is the process of performing automated and human evaluation at the same time. Sometimes, automated, human evaluation, and user expert testing are also referred to as hybrid testing. It helps to compare and improve the multiple testing results, simultaneously.

To the result of the background study, the majority of the previous studies proposed web accessibility evaluation techniques using several existing automated tools. AlMeraj et al. [5] present the evaluation result for higher education websites of Kuwait using three automated website accessibility testing tools, such as AChecker [28], total validator [29], and WAVE [30]. It shows that, of 48 websites, only 11 websites were found accessible through the evaluation tools. Ali [19] investigated 87 educational websites of the UAE through automatic testing tools: A-CHECKER, CYNTHIA SAYS [31], and TAW [32]. Results indicated that only 5 websites were accessible through the automated evaluation tools. Zare et al. analyzed 51 medical science websites of Iran for identifying the accessibility of these websites [33]. They suggested that, using the ‘WEB accessibility’ automated testing protocol, none of the websites were effective and suggested continuing the testing with other existing automatic tools. Król et al. used WAVE to analyze 91 hospital websites of Poland and found that none of the websites passed accessibility testing using this particular testing tool [34]. Alsaeedi et al. [35] and Kuppusamy et al. [36] investigated 6 Saudi Arabian university websites and 25 Indian higher educational institution websites. They used WAVE, SiteImprove [37], and AChecker and found that most websites have low accessibility scores, and few of them passed the accessibility testing. Alshamari et al. [38] investigated 3 E-commerce websites of Saudi Arabia through 4 accessibility testing tools: Achecker, TAW, MAUVE [39] and FAE [40]. They found that navigation, readability, input assistance and timing are the common accessibility problems. However, the majority of the existing literature investigated private websites. In 2017, Ismail et al. investigated 33 general administrative departments websites of India using some automated tools [41]. They employed AChecker, Cynthia Says, Tenon [42], WAVE, Mauve, and Hera [43], free web testing protocols to perform the analysis. They found that most of the government websites had poor accessibility, with several errors.

After reviewing the most recent studies, we found some past studies regarding education, healthcare, and E-commerce websites [44]. Due to the COVID-19 pandemic, a significant number of people are engaging in the COVID-19 portal for information. Nowadays, COVID-19 vaccine-related websites are more popular among the people of the different countries of the world. Therefore, evaluating COVID-19 vaccine government websites is crucial. Focusing on this issue, Alismail et al. [21] evaluated 54 COVID-19 vaccination registration websites of the US using WAVE, AChecker, and SortSite [45] evaluation tools. They found that only nine websites were accessible, where two came from WAVE and SortSite and five came from AChecker automated tools.

However, to the best of the author’s knowledge, none of the studies performed the evaluation process on government COVID-19 vaccine-related websites, even though the importance of ensuring accessibility is beyond question. The majority of the existing approaches only focused on the automated tools, yet, other testing aspects are also significant and can improve the evaluation result. Therefore, in this study, we evaluated COVID-19 government vaccine websites through the hybrid technique. We implemented both automated tools (Mauve, Nibbler, WAVE, and WEB accessibility) and human observation through questionnaires. Additionally, this analysis will help to understand the effectiveness of the automated tools and human validation.

## 3. Materials and Methods

**Figure 2:**
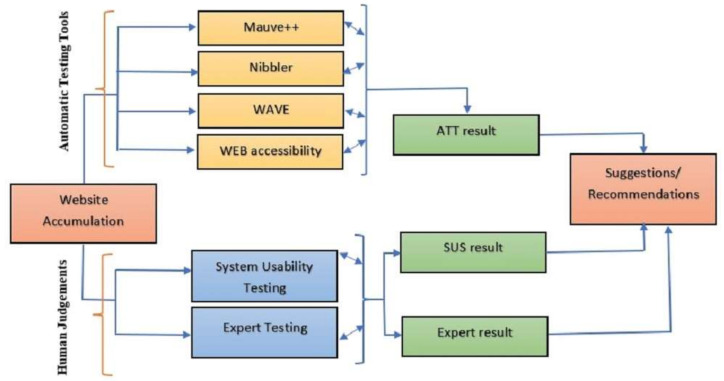
Workflow diagram.

This study presents the investigation of SARS-CoV-2 related government websites to understand their accessibility for people with disabilities. The government websites provide valuable information such as safety guidelines, COVID-19 symptoms, the total number of active cases, the vaccine registration process, etc. From this perspective, web technologists and previous researchers proposed several works for web accessibility and directed for future research. Therefore, in this work, we employed automatic web accessibility testing tools to investigate the most common accessibility problems of the COVID-19 vaccine information-related websites and to identify the accessibility of these sites. Figure 2 shows the workflow diagram of the investigation process of the COVID-19 vaccine information websites through automated tools.

### 3.1. Website Accumulation

**Figure 3:**
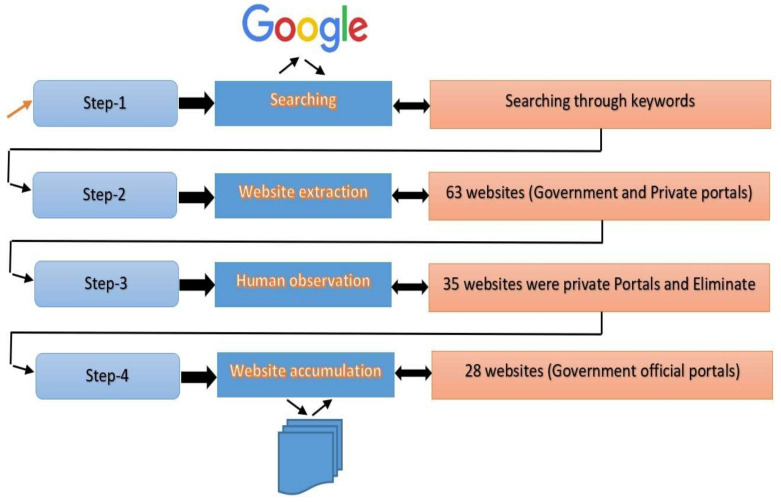
Websites selection for accessibility evaluation.

In this study, we considered websites from two groups of the country: group-1: Twelve European countries (Estonia, France, Greece, Ireland, Latvia, Luxembourg, Netherlands, Iceland, Austria, Switzerland, Croatia, Germany), and group-2: Eight Asian countries (India, the Philippine, Jordan, UAE, Kyrgyzstan, Maldives, Hong Kong, and Qatar). Initially, 63 websites have been found for both governmental and private institutions (Figure 3, step-2). The prime goal of this study was to investigate the official government websites only. Therefore, we eliminate all the private organization websites from the investigation. After eliminating the private websites, a total of 21 websites were found as official government websites and were considered for our evaluation, as shown in Figure 3. The considered websites are shown in Table 1 with their country, region and page URL.

### 3.2. Automatic Accessibility Testing Protocols

#### 3.2.1. Mauve++ Tool

**Figure 4:**
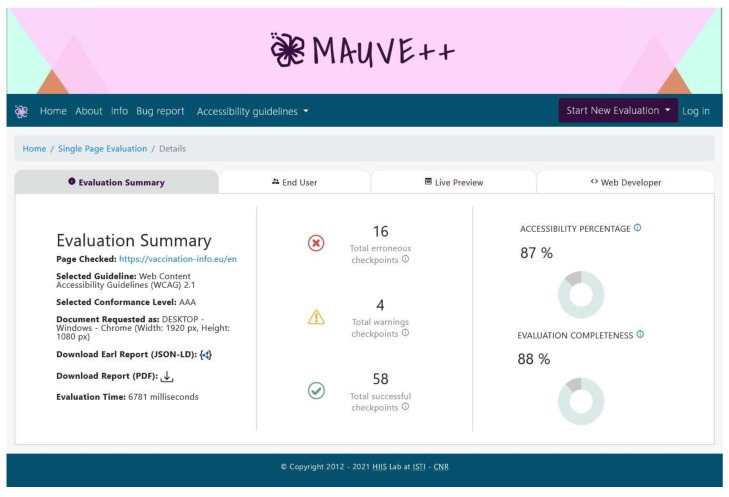
Screenshot of Mauve web accessibility testing tool.

Multiguideline Accessibility Usability Validation Environment (MAUVE) is an environment for web content accessibility evaluation according to WCAG standards [39]. The researchers of HIIS Lab developed and provided it for the community as an open access testing environment. Generally, it works for usability criteria identification to improve the website navigation for physically disabled people, specifically focusing on the end-user demands. It helps to determine website accessibility through WCAG 1.0, WCAG 2.0, and WCAG 2.1 standards. It focuses on three conformance levels: A, AA, and AAA to test websites. Conformance levels represent a set of requirements that must be fulfilled to make content available online. According to the WCAG standards, there are three conformance levels; every conformance level has different guidelines or requirements. Therefore, before publishing the content online, authorities should focus on the conformance requirements and should satisfy all the conformance levels (A, AA, AAA) to make it accessible. MAUVE++ automatic accessibility testing tool checks against all three-conformance level requirements during the evaluation process of websites. MAUVE allows running the software in a browser plugin, desktop, smartphone, iPad, tablet, etc. It is possible to download the result in JSON format. It generates the report through the total number of successful checkpoints, warning checkpoints, and erroneous checkpoints. The overall accessibility percentage generated is based on the ratio of the number of checkpoints passed and the number of checkpoints tested. Generally, it provides the error based on HTML and CSS rules violation according to the WCAG guidelines. It can be tested on both single-page and dynamic page websites simultaneously without any interruption. Figure 4 shows the web accessibility result of the European vaccination information website through the Mauve++ testing environment.

#### 3.2.2. Nibbler Tool

**Figure 5:**
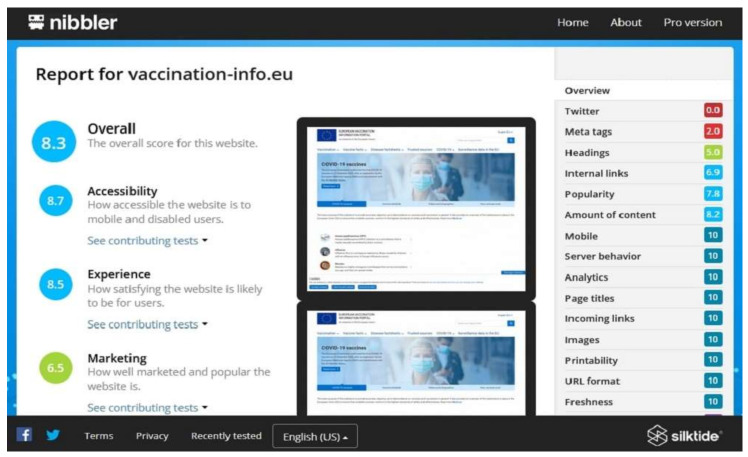
Screenshot of Nibbler web accessibility testing tool.

Nibbler is a free website accessibility testing tool that accesses the website URL and generates the accessibility result [46]. In a word, Nibbler is a ‘bot’ or ‘automated computer program that works by looking at the domain name to find the linked web pages. It calculates the overall accessibility score on a 10-points scale based on four key prototypes (accessibility, experience, marketing, and technology). Each prototype analyzes several aspects such as code quality, headings, internal links, mobiles, page titles, URL format, amount of content, Facebook page, freshness, images, popularity, printability, server behavior, Twitter, analytics, domain age, incoming links, meta tags, and social interest to determine the overall accessibility score. Figure 5 shows the screenshot of the Nibbler tool for the European Union vaccination information website.

#### 3.2.3. WAVE Tool

**Figure 6:**
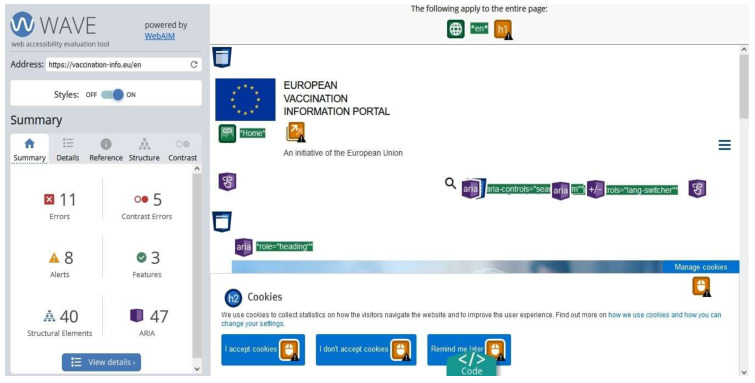
Screenshot of WAVE Web accessibility testing tool (view a).

**Figure 7:**
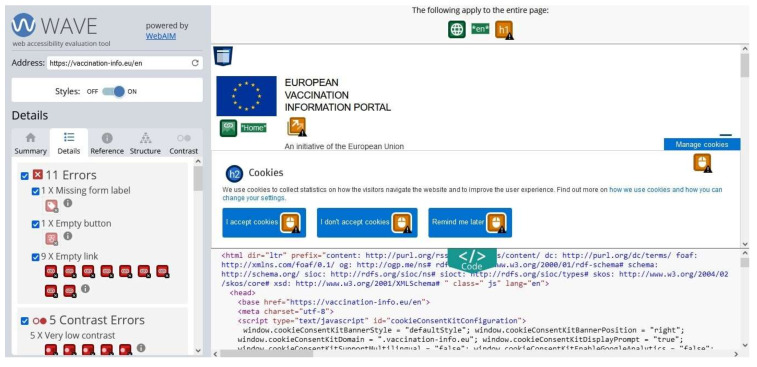
Screenshot of WAVE Web accessibility testing tool (view b).

WAVE is a free, open access, single-page web accessibility checker [30]. It works by checking the number of errors, contrast errors, alerts, features, structural element issues, accessible rich internet application (ARIA), and text size in terms of normal and large context. WebAIM developed WAVE for both website and plugin versions. It performs following the entire WCAG guidelines, including, WCAG 1.0, WCAG 2.0, and WCAG 2.1 standards. Figure 6 and Figure 7 shows the view of the WAVE test result for the European Union vaccination website. It summarizes the entire problems as shown in Figure 6 (view a) on the left of the screen. The detail section presents all the violations in detail and provides potential solutions via the source code as shown in Figure 7 (view b).

#### 3.2.4. WEB Accessibility Tool

**Figure 8:**
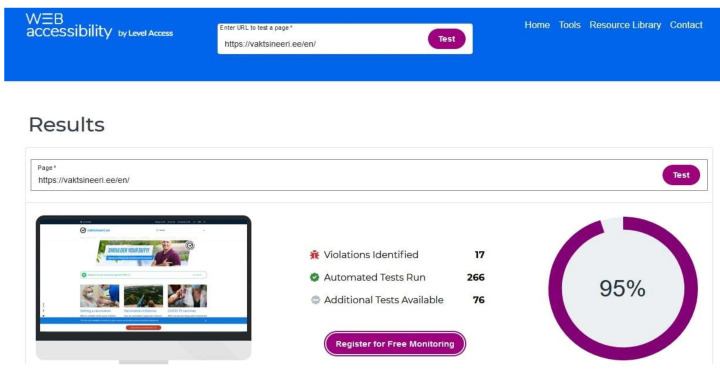
Screenshot of WEB accessibility tool.

WEB accessibility is another popular website accessibility testing tool that highlights the graphical view of the original website [47]. It is free, open-source software and is publicly available for the entire browser (Chrome, Firefox, etc.) as a testing medium. It works by calculating accessibility percentage through compliance score, number of identified violations, number of automated tests, and suggests the additional test that should be carried out. Additionally, it provides details about identified violations with their severity level. Figure 8 shows the result of a vaccination information website for Estonia by WEB accessibility tool with a 95% compliance score.

### 3.3. Questionnaire-Based Human Observation

According to the past research [3,5,21], automatic web accessibility testing tools are effective. Though automatic tools have high effective measurement, still, several issues are unrevealed by these tools. Thus, we perform human observation through two testing systems: system usability testing and expert testing to validate the accessibility issues.

#### 3.3.1. System Usability Testing

Through System Usability Scale (SUS), we can understand the effectiveness of the websites for a particular group of people [48]. To do the system usability testing, we invite few foreign volunteers to measure the usability of the websites and provide their feedback according to ten questions. We set the system usability measuring scale from Strongly Disagree (1) to Strongly Agree (5) to provide the usability feedback. Table 2 shows the example of questionnaire-based system usability testing format that have used in this research. The detailed result has shown in Section 4.

All the volunteers who participated in this testing were university third-year (of four-year studies) bachelor students from the computer science and engineering department of Jahangirnagar University, Bangladesh. Every student had completed the ‘User Interface Design’ course. Thus, they were capable of observing the websites according to the questionnaire. The number of students was 10, including 4 female and 6 male students between 19 and 22 years of age. To investigate 21 websites, we arranged online participation and shared questionnaires and website information with the students. To distribute the work, we classified 10 students into two groups: group-1 belonged to 6 students, and group-2 belonged to 4 students. Group-1 was responsible for evaluating European websites, and group-2 was responsible for the evaluation of Asian websites. In group-1, we asked 5 students to evaluate 2 websites each, and 1 student was assigned to investigate 3 websites. Besides, in group-2, 4 students were responsible for testing 2 websites each. The calculated average investigation time was 15 min/website. The investigation process in detail is shown in Table 3.

#### 3.3.2. Expert Testing

Expert testing is the process of human testing that identifies the existing problems of a particular website and makes recommendations to upgrade the inaccessible functions. To do the expert testing, we set twelve questions with a yes/no answer option and sent them to the foreign experts to provide their responses according to their observation. For the expert testing, four experts had been invited in this research to validate the selected websites. The occupation of the experts includes an associate professor, an assistant professor, a senior lecturer, and a Ph.D. student from the department of electrical engineering and information systems, University of Pannonia, Hungary, and department of computer science and engineering, Jahangirnagar University, Bangladesh. All the experts validated 21 websites and provided their findings. Every expert had least 5 years of research experience in web accessibility, multimedia design, human–computer interaction, and web informatics fields. Table 4 shows the example of questionnaire based expert testing format that have used in this research. The detailed result is shown in Section 4.

## 4. Test Results

This section describes the test results in detail based on the investigation we have performed in the period of June–August 2021. During this period, people all over the world were seeking vaccine information more actively. Therefore, identifying the accessibility issues of the websites significantly increased during that time.

### 4.1. Result of Mauve++ Test Tool

To identify the accessibility of the selected COVID-19 government vaccination websites, here, we employed a Mauve test based on WCAG 2.0 and WCAG 2.1 standards according to the POUR principles (P-Perceivable, O-Operable, U-Understandable, and R-Robust). Based on the error rate, the overall performance is calculated for conformance level AAA, as shown in Table 5.

To evaluate the performance or website quality according to accessibility testing results, we classified the website accessibility analysis result into three categories: High, Medium, and Low. The error rate (%) 2qs calculated from the total number of errors found during the test. Generally, fewer errors represent high-performance websites; a higher number of errors represents the low performance of the websites. Therefore, to evaluate the error rate (%), a significance level was taken into consideration. From our empirical observation and researcher opinion, we considered the significance level α = 0.05 and α = 0.09 to evaluate the website performance. Those websites were classified under α <= 0.05, considered as ‘High’ quality websites. If websites were classified under α > 0.05 to <=0.09, then we considered them to be ‘Medium’ quality websites. Furthermore, under α > 0.09 significance level, websites were considered to be ‘Low’ quality websites.

Table 4 shows that, according to WCAG 2.0 guidelines, among 21 government websites from different countries (European and non-European/Asian), only 5 websites were classified under significance level (α ≤ 0.05), where four were from Europe, and one was from Asia. These five websites were High-quality websites according to WCAG 2.0 guidelines. After evaluating the results under α > 0.05 to ≤ 0.09 significance level, eight websites were Medium-quality websites, from the European and non-European countries (six from Europe, and two from Asia). Seven websites were Low-quality websites, two from Europe and five from Asia. For WCAG 2.1 guidelines, only two websites were retrieved under the significance level α and marked as High-performance websites that belong to the Europe and Asia region. However, five medium-quality websites were found, three from Europe and two from Asia; 13 Low-quality websites were from Europe (8) and Asian countries (5).

Additionally, through the MAUVE testing tool, only 4% of the websites (1 out of 21) passed the MAUVE test without any error according to WCAG 2.0 and WCAG 2.1 guidelines. For WCAG 2.0 standards, the remaining website failed, with an average of seven errors. Moreover, for WCAG 2.1 standards, all the websites failed, with an average error of nine. However, for the WCAG 2.1 guideline, the average error of Europe and Asian countries was comparatively higher than the WCAG 2.0 guideline. In both WCAG 2.0 and WCAG 2.1 guidelines, the number of unavailable websites was 1 (WID-12).

Table A1 and Table A2 show the accessibility errors of our targeted websites in detail for WCAG 2.0 and WCAG 2.1 standards. The related principles and the corresponding success criteria are shown in the tables. Generally, the MAUVE testing tool generates errors by following the WCAG criteria. Among several violations detected by the Mauve++, size and position, Internal links, CSS, ARIA landmarks, and alignment were the most frequent violations for the majority of the websites. This means that websites were larger than usual, positions were not appropriate, and internal links were not working perfectly, or were not distinguishable from the text. In addition, ARIA landmarks describe various issues, including inappropriate names, heading, etc. These issues cause difficulties for disabled users, specifically, those who use screen readers, braille, or text reader.

**Figure 9:**
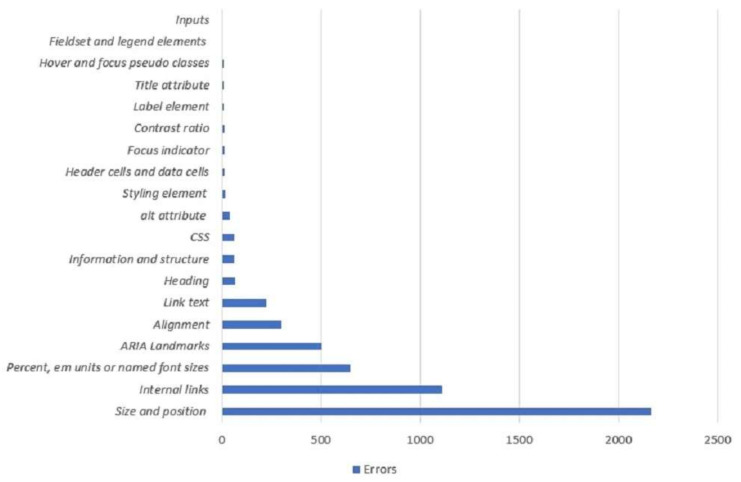
Frequent violations identified by Mauve++ for WCAG 2.0, 2.1.

Furthermore, Figure 9 summarizes all the accessibility errors detected by Mauve++. From this figure, it is clear that, for 21 websites, issues associated with size and position arose 2166 times. Issues with size and position implied that many websites were not focusing on their websites’ length. It is the prime issue for lack of support in several systems. Surprisingly, problems associated with internal links arose 1113 times, which meant that internal links might be inappropriate, unavailable or damaged. Some other issues with ARIA landmarks appeared 506 times, alignment appeared 303 times, and link text issues arose 224 times. ARIA landmarks allow assistive technology to understand and navigate the page or content semantically. Alignment issues refer to the fact that the text or content of the website is not aligned correctly. It causes difficulties for blind people who use assistive technology. Besides, the link text issues appear for missing text in the available link.

### 4.2. Result of Nibbler Tool

Nibbler is an automatic web accessibility testing tool used to evaluate the website’s accessibility. Table 6 shows the Nibbler test result of the selected government websites. We evaluated the pages according to their overall score. The evaluation fell into three assessments categories: High, Medium and Low. Websites with an overall score between 10 and 9.50 are considered to be high-quality websites. If the pages had a score between 9.49 to 9.0, they were considered to be Medium-quality websites. Pages with an accessibility score between 8.99 to 0 were considered to be Low-quality websites. One website achieved a 9.70 accessibility score (WID-11.0), indicating a High-quality website. Three websites were determined to be Medium-quality websites; seventeen websites were low accessibility websites. During testing, the Nibbler tool was able to generate the test report for all the websites.

Table A3, shows the summary of accessibility errors obtained from the Nibbler tool for 21 targeted websites. Similar to Mauve++ and WAVE tools, Nibbler also provides some suggestions for associated errors. For example, on the website of Estonia, errors with incoming and internal links appeared four times, issues with popularity; amount of content appeared two times each. On the websites of France, issues with popularity, server behavior, meta tags, and amount of content appeared two times each. Problems with internal links appeared four times. These issues occur for inappropriate link placements, insufficient information, irrelevant tags, limited server ability, etc. However, the conducted experiment showed that these types of errors were common in all the website homepages. Moreover, Table 8 concludes that each country’s websites had different issues that cause several problems associated with accessibility.

**Figure 10:**
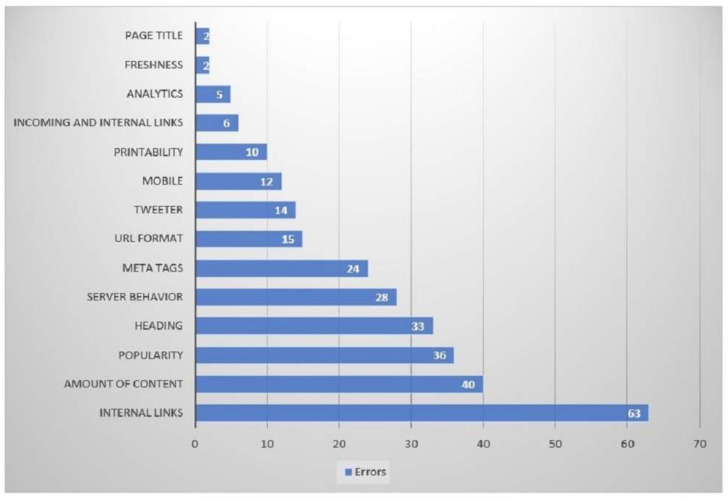
Frequently appeared violations identified by Nibbler.

Figure 10 shows the number of errors found by the Nibbler tool. Issues with internal links appeared 63 times, which meant that internal links did not work correctly or had some errors. Then, issues with the amount of content arose 40 times. This refers to the amount of content in the websites that was not sufficient or that was not updated. Few errors with popularity issues appeared 36 times; this indicates that the websites were not accessible for certain people. Some issues with heading, server behavior, meta tags, and URL format detected by the Nibbler tool appeared 33, 28, 24, and 15 times, respectively. Some issues with Twitter, mobile, printability, incoming links, analytics, freshness, and page title were also found, but these issues were not frequent.

### 4.3. Result of WAVE Tool

To evaluate the website accessibility and quality, we employed an automatic web accessibility testing tool named ‘WAVE’. WAVE evaluates the websites based on six categories, features, errors, alerts, contrast errors, structural elements, and ARIA. After experimenting, the analysis result revealed that (Table 7), among 21 websites, only 3 websites (WID-1.0, WID-5.0, WID-19.0) passed the accessibility test. The medium performance website had the lowest number of errors, two (WID-6.0, WID-8.0 and WID-20.0). Among 21 websites, 6 websites had fewer than the average number of errors, six. Four websites had six to eleven errors; five websites (WID-14.0, WID-13.0, WID-11.0, WID-18.0, and WID-15.0) had a total of 25, 29, 31, 47, 59 errors, respectively. Considering the contrast error result, sometimes significant contrast errors might affect content viewing and reading. The majority of the websites (71%) had contrast errors; the maximum contrast error was 100, found for WID-14.0. Some websites were free from contrast errors, which were around 28%.

Table A4 reports the accessibility errors detected by the WAVE for our targeted websites. It should be highlighted that the Mauve++ test detected more errors than WAVE. However, the WAVE testing tool helped to find some specific issues. For example, WAVE provides several issues associated with links, texts, images, and languages. For instance, broken skip links, empty links, missing alternative text, linked image missing, and language missing. These error details help to understand the exact problem: ‘empty link’ meant that the link had no information; ‘linked image missing’ meant that the image was missing in the given link.

**Figure 11:**
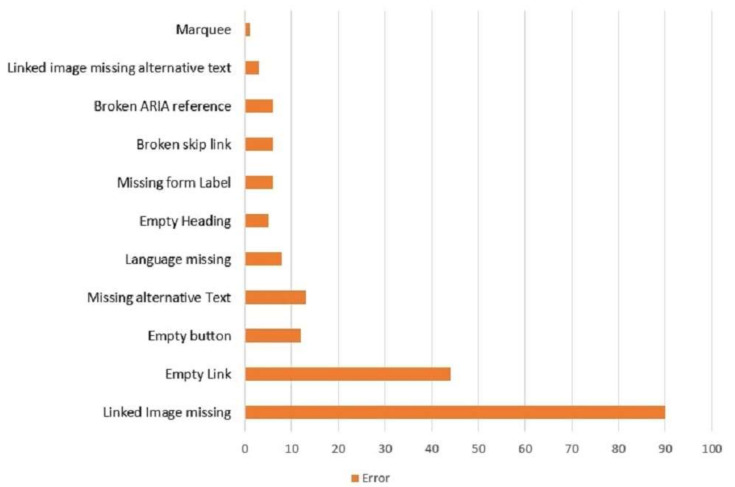
The most common errors for WAVE tool.

Figure 11 shows the most frequent violations or errors found by the WAVE. The maximum number of the errors found for linked image missing was 60; empty link errors was 44. Linked image missing meant that the image was not present in the link; empty link error meant that the webpage did not contain text. Empty button, missing alternative text, and language missing accounted for 12, 13, and 8 errors in total, respectively. An empty button means that the button is empty or has no text value. Missing alternative text means the alternative text is not present, and language missing refers to some language barriers present, such as words, sentences, etc. Empty heading, missing form label, broken skip link, and broken ARIA reference appeared with six and five errors, respectively. Empty heading refers to a missing heading. Missing a form label meant that the form control did not have a corresponding label. A few errors appeared for linked image missing alternative text (3) and missing or uninformative page title (1). This error might cause some inaccessibility to the website user. Therefore, web developers should ensure that all the content is accessible to users and should follow WCAG guidelines.

### 4.4. Result of WEB Accessibility Tool

The evaluation results of the WEB accessibility validation tool are presented in Table 8 with both compliance score and number of violations. Previous studies have considered the compliance score for evaluating the websites. Generally, a compliance score does not always depend on a specific attribute. In some cases, websites have a high compliance score, but also have several instances of violation; sometimes, the scenario is reversed. Therefore, this research did not consider compliance scores. To evaluate the COVID-19 associated government websites, we considered the violations/errors only. The highest number of errors was for WID-7.0, with an average of 104. The lowest number of violations was for WID-4.0, WID-5.0, WID-11.0, WID-20.0, with an average violation of 1. Among 21 websites, only 2 websites (WID-2.0 and WID-12.0) passed the accessibility testing with 0 violations. The majority of the websites recorded a significant number of violations, ranging between 2 and 104.

The accessibility errors determined by WEB accessibility for the official government websites from different parts of Europe and Asia, are shown in Table A5. For example, on the website of Estonia, 15 images did not have an alternative text. On the website of Greece, four links were not meaningful with the context. There were several other issues associated with the context, headings, and labels.

**Figure 12:**
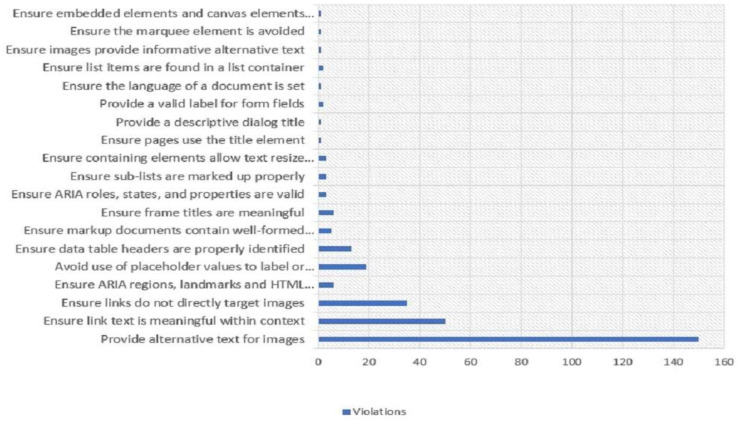
Violations identified by WEB accessibility tool.

The WEB accessibility tool retrieved several violations during the testing of the COVID-19 vaccine websites. Figure 12 summarizes the entire violations of the websites. Violation with ‘providing alternative text for images’ appeared 172 times on 28 websites. ‘Ensure link text is meaningful within context’ appeared 44 times, and ‘Ensure links do not directly target images’ appeared 35 times. In addition, violations associated with the error ‘Ensure ARIA regions, landmarks, and HTML sections are identifiable’ and ‘Avoid placeholder values to label or explain input’ appeared between 21 to 25 times.

### 4.5. Result of System Usability Testing and Expert Testing

To calculate the SUS score [38] of the targeted websites from the questionnaire-based user feedback, first, we calculated the score of the odd number of questions, $Qscore\left(Odd\right)=Xn-1;\;\mathrm{where}\;n=1,3,5,7,9$ and then we computed the score of the even number of questions, $Qscore\left(Even\right)=5-Xn;\;\mathrm{where}\;n=2,4,6,8,10$. Then, we calculated the SUS score through Equation (1).

(1) $$SUS_{score}=\sum_{n=1,3,5,7,9}\left(Xn-1\right)\;*\;2.5+\sum_{n=2,4,6,8,10}\left(5-Xn\right)\;*\;2.5$$

To determine the quality of the websites, we set six quality parameters based on the SUS score, shown in Table 10. Based on the quality parameter, we evaluated the websites. Table 9 shows the SUS score of each website with evaluation remarks. 

After evaluating the websites through the SUS score, only 14% of websites were classified as ‘Excellent’ websites (WID-12, WID-15, and WID-19); 9% of websites (WID-2.0, WID-3) were found to be the ‘Best’ quality websites. Furthermore, only 28% of websites (WID-1, WID-5, WID-6, WID-7, WID-14, WID-20) were ‘Good’ quality websites. These websites were accessible to people who have some disabilities. However, the ratio of these accessible websites’ was not sufficient to ensure that all the websites were accessible. Besides, the rest of the websites had no adequate functionality or accessibility; website operators must take websites’ accessibility into consideration. For instance, fair, poor, and worst websites’ ratio was 19%, 19%, and 9%, respectively. This result suggests that a significant number of the website’s accessibility issues should be under consideration for further improvement.

The expert investigation results are displayed in detail in Table 11. They show that the majority of the websites were not accessible for people who had vision problems or moving disabilities. On average, 70% of websites were not accessible for people who had a low vision concern. Some websites had keyboarded/TAB key accessibility, but not for all the buttons or sub menus, making the website inaccessible. Few of the websites had a color deficiency; it was hard to separate the link and buttons. Surprisingly, none of the websites had manual font size function, which makes the websites difficult for people who have low vision concerns. Additionally, only a few websites were available in English; a few were not responsive. With the automatic tools, we were unable to detect these issues that sometimes fail to produce the appropriate research output. Thus, human observation is effective to identify the unreported problems of automatic tools.

## 5. Evaluation Results 

Ensuring adequate access to online content is a primary objective in the field of information technology. Sik Lanyi et al. have also added that, so far, the majority of websites are not accessible for people with disabilities [3]. Therefore, in this study, we tested 21 official government COVID-19 vaccine information portals from various countries in Europe and Asia. This study aimed to understand the current insights of web accessibility and to identify the frequent violations. In this global pandemic, official government websites were valuable, reliable, and trusted sources for COVID-19 vaccine information for people, including those with disabilities. Thus, compliance checking of these COVID-19 vaccine information portals against the WCAG standards is crucial to make the web accessible and understandable for both people with special needs and non-disabled users. Therefore, we used four automatic accessibility checking tools and human observation techniques to perform the testing process. The automatic testing result revealed that the majority of the websites had accessibility issues. Additionally, human observation suggested some improvements to make the websites accessible.

**Figure 13:**
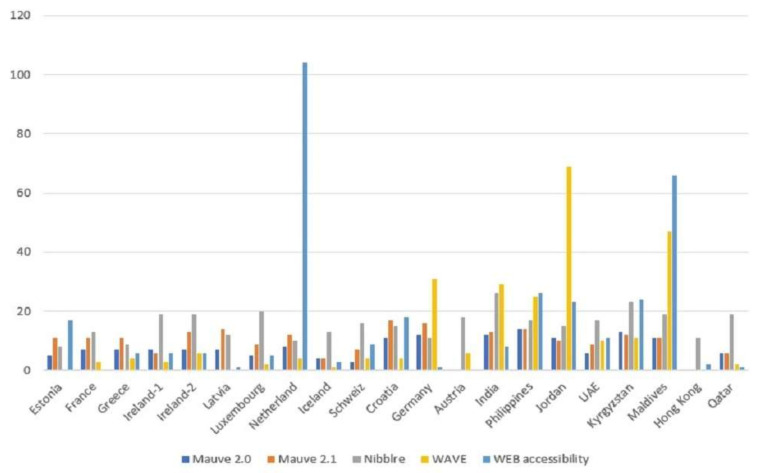
Error ratio of each individual country through automatic tools.

According to our empirical observation (shown in Table 12), Asian countries’ websites had a higher number of errors on average compared to websites operated by European countries. From our observation, the cause for higher numbers of errors in Asian countries is because most of the Asian universities lack educational materials for digital accessibility. Therefore, graduate students were unaware of accessible development, its importance, and related consequences. In contrast, European countries have courses regarding digital accessibility that help the student to understand accessibility and implement it at the very beginning of their carrier. Therefore, we rejected the first part of the H1, RQ1. Furthermore, Figure 13 shows the ratio of errors for each country for four automatic testing tools. Through Mauve testing, for 2.0 standards, the maximum number of errors was 14, found on the website of the Philippines. For 2.1 standards, the maximum number of errors was 17, found on the website of Croatia. In the WAVE testing tool, the highest number of errors was 69, found for Jordan. Following Jordan, Maldives had 47 errors, which is the second-highest number of errors. If we look at the results of Nibbler, the maximum error rate was 26, found on the website of India. We found 66 errors on the Maldives website, which is the highest among others (WEB accessibility). Therefore, it is obvious that, in the accessibility results, there were some differences across the regions, continents or countries; we accepted the last part of H1, RQ1. This result also revealed that web developers from Asia should be more aware of accessibility criteria.

Table 13 shows that the average SUS score of European and Asian countries’ websites were 66.92 and 56.25, respectively. Furthermore, based on the expert questionnaires (shown in Table 4), the total average score of European and Asian countries was 0.68 and 0.71, respectively, as shown in Table 14. Although the average number of SUS score, and the total number of errors, were higher on the Asian websites, no significant difference was found. Additionally, there was no significant difference in the score of each question. Therefore, we accepted the H2, RQ2.

According to the automatic evaluation results, few websites were accessible. However, after going through questionnaire-based human judgment (system usability and expert testing), we found some inaccessible functionalities in these websites. Regarding the SUS testing, we could determine the quality of each website (Table 10), which is not possible to determine through any automatic testing tool. Through the automatic tool, it was only possible to identify whether the website had passed or failed the accessibility testing. In most cases, the existing automatic tools had limitations for incorporating all the WCAG guidelines. Therefore, a few accessibility issues were not possible to detect by the automated tools that directed future research. However, regarding the expert testing, neither of the websites had manual font resize functionality, which could be a problem for people with poor vision. Some websites had no accessibility option when using the TAB key. An excellent example could be WID-18, having no movement function with the TAB key. This website was not accessible for people who have moving disabilities. Some websites were not in English, and there were no translation options, e.g., WID-9 had no English version or translation options. Therefore, sometimes human judgment helps to find unexplored issues that are not possible through the automatic tools. Thus, there were a few differences between automatic and human judgment results; we accepted H3, RQ3.

Furthermore, Table 15 shows the error types, associated principles, and their number of occurrences. Among the four principles, the highest number of errors was for the first principle. Therefore, we accepted H4, RQ4. All the websites failed to add ‘size and position’, ‘internal links’, ‘linked image missing’, and ‘alternative text’ for images. For instance, the website of India had 1219 issues with size and position and 594 issues with internal links. On the website of the Maldives, 35 links had no images or the images were missing. The website of the Netherlands had 103 images without alternative text, which is higher than other websites (a result of WEB accessibility). Additionally, all the web pages had accessibility issues, such as no added text for links to describe the target links. A proper description of the links could help the people who are using a screen-reader or text browser. The webpage of Germany had 15 empty links, which is higher than any other webpages.

Based on our measurements, we have found that more testing tools need to check the accessibility of vaccination-related websites and even need to be supplemented with expert testing. So, our thesis group is:

**Thesis** **group** **1** **(T1).** *There are differences between European and Asian counties’ websites accessibility according to their average number of errors. Moreover, there is a difference when focusing on the website of each country.*

**Thesis** **group** **2** **(T2).** *There is no significant difference between websites from the European and Asian, considering questioner based human judgment results.*

**Thesis** **group** **3** **(T3).** *There is a significant difference in the result of automatic testing and questioner based human judgment.*

**Thesis** **group** **4** **(T4).** *The first principle of WCAG 2.0 and WCAG 2.1 is the most frequent violation principle, considering four automatic testing tools.*

## 6. Discussion

Past research revealed that individuals with disabilities have different accessibility issues. Thus, the current internet platform or web-based portals were not completely accessible for people with disabilities, in some cases, not even for non-disabled users [23]. Therefore, this research performed evaluation on the COVID-19 vaccine information websites to investigate their accessibility status. The consequences of the inaccessibility of the COVID-19 vaccine information portals might hamper both social and economic life. For instance, COVID-19 brings restrictions on the border. Without proper vaccination, it’s impossible to get treatment, travel, etc. Some countries’ vaccination regulations and acceptance were different from others. Thus, without proper vaccine information, it was not possible to serve consistent services for education, business, etc. To access the vaccine information, people frequently used the official COVID-19 vaccine information portals. Therefore, investigation and identification of accessibility issues of COVID-19 vaccine information portals is an open issue for the web researcher.

The automatic testing and human observation results revealed that a significant number of the COVID-19 information websites were not free from accessibility issues. The prime reason is that a majority of the websites did not follow the compliance standards of web content accessibility guidelines. It also indicated that countries were did not consider accessibility issues during the designing and development phase. Frequently, web developers ignore accessibility guidelines for website development. Therefore, our present research emphasized that people with disabilities should be taken into consideration during website development, as they take part in the development of the economy. After evaluating 21 websites from different parts of Europe and Asia, several accessibility problems were identified in this study through four automatic tools and human judgment. These indicated that all the accessibility issues could be easily fixed. Based on the accessibility testing report generated by Mauve++, Nibbler, WAVE, and WEB accessibility, we encourage designers and developers to follow the WCAG standards issued by the W3C. Some of the frequent accessibility violation guidelines are addressed during the testing, which requires additional attention by web developers during development.

Guideline 1.4. Distinguishable: This guideline is under the perceivable principle and was the most frequently violated guideline reported by Mauve++ during the COVID-19 vaccine information websites investigation. The Mauve++ automatic accessibility testing tool generated this report and revealed that 135 issues arose under this guideline during the website’s investigation. The report using the WCAG 2.0 and WCAG 2.1 version guidelines indicated that the majority of the websites had no clear visual representation or had issues with color, audio quality, text size, contrast ratio, images of texts, and non-text contrast. More attention is needed to improve the visual representation and audio quality to allow consistent services for screen reading application users and to allow modification based on individual needs. Being unable to resolve these issues causes a barrier for people with visual impairment in accessing the information properly.

Guideline 3.2. Predictable: The predictable guideline is under the understandable principle at conformance levels A, AA, and AAA. It was the most violated guideline detected by Nibble automatic testing tool. Nibbler identified 29 issues from this particular guideline, which was the maximum in investigated COVID-19 vaccine information websites. Following this guideline, it is possible to design proper user interface components and to ensure equal service for people with disabilities and non-disabled users.

Guideline 2.4. Navigable: Navigability is an important guideline for accessibility under the operable principle at conformance levels A, AA, and AAA. It was the most frequently violated guideline, with 30 issues identified by WAVE during the investigation of COVID-19 vaccine information websites. This guideline aims to ensure consistent navigation to find content and to determine its exact location on the website. The report raised issues with the page title, heading, labels, and link problems. These issues indicated that a majority of the websites lack concern about accessibility guidelines, which would ensure accessible content to people with disabilities. Improper title and heading labels cause inaccessibility and impede the identification of the exact information. These issues also cause a barrier to navigating the websites properly and increase dissatisfaction for people with special needs or who require assistive technology.

Guideline 1.1. Text alternatives: This guideline is under the perceivable principle and the frequently violated guideline at conformance level A. The WEB accessibility testing tool detected 17 issues associated with this guideline on 21 official government COVID-19 vaccine information portals. These findings indicate that the majority of the websites had issues with incorporating text alternatives for non-text content. By ensuring text alternatives for non-text content, it is possible to help people with visual challenges. For instance, without text alternatives of images, people with visual impairment would not be able to understand the content of the image. Therefore, it helps visually impaired people and users who use braille or other assistive technology for accessing information. It also helps people with cognitive disabilities who have difficulty understanding the images and who need simpler language.

Several issues are responsible for these accessibility errors. Generally, due to cost-effectiveness, developers ignore accessibility testing (automatic, human testing) before publishing the website for the user. Frequently, they use redundant templates instead of considering updated development tools. In addition, most of the developers prefer to choose the most common development tools (HTML, PHP) and do not consider advanced tools like JavaScript, React.js, etc. Lack of monitoring systems also increases the number of inaccessible websites.

Moreover, the findings suggest that the evaluated websites had limited focus on the usage of color, contrast, audio component, text size, resizing, spacing, text alternatives, image alternatives, page title, heading, levels, link purpose, user interface, and visual components. Although these issues were frequent, some additional issues also needed to be considered, such as adaptability, readability, and compatibility. Similar to [21], we want to emphasize that, with automated accessibility testing, user testing is highly beneficial, as it allows for involving people with special needs. They can provide the actual feedback regarding their capabilities. Additionally, assistive technology should be incorporated during testing.

From our observations, the investigation results imply that authorities should pay more attention to accessibility issues during the design and development phase. More accessibility evaluation processes should be carried out in the future, as websites are updated constantly. Therefore, frequent evaluation will help to identify and solve the emerging issues. In addition, further training should be organized for web designers, web developers, and other associated authorities to increase the awareness and importance of accessibility to make internet platforms accessible.

## 7. Suggestions for Future Improvements

This section presents some suggestions to improve the accessibility of web pages. All the suggestions are regarding automatic web accessibility testing, system usability, and expert testing results. To develop accessible web platforms, the following suggestions should be incorporated during the development phase.

**Image Alternatives:** Image alternatives is the process of representing text information through non-textual content (images, videos, animations, etc.). By ensuring alternative representation techniques, it would be barrier-free for users with disabilities.

**Text Alternatives:** Text alternatives is the process of representing the non-textual (images, videos, animations, etc.) information through some textual information or description. Lack of a text alternative’s function makes the websites difficult to understand for users with disabilities, especially for those who use screen readers to consume the information.

**Text-size and Color Contrast:** Color contrast is one of the most important accessibility criteria for making websites or online content accessible to people with disabilities, as different people have their demands. Several websites use text-size and color contrast adjustment functionalities, but the majority of the websites have no focus on this specific requirement. Thus, website administrators and developers should use these functions.

**Multilingual accessibility:** For web page development, each web page should have its native language version and the English translated version. English translation helps people speaking different languages to access the website. During translation from one language to another, precise translation must be of great emphasis to reduce the barrier of accessibility.

**Design Accessibility:** Websites have various statistical and graphical representations; an unorganized design structure could result in an inaccessible website. Graphical representation and website organization should be clear and easy to understand.

**Proper title and subtitle:** Documents or pages should have an appropriate title and subtitle (if required based on demand) to navigate the page appropriately for users who rely on a screen reader.

**Links or hyperlinks placement:** In the document or content, links or hyperlinks should be placed correctly so that people with disabilities can easily understand them. Links should be placed with proper attributes. Thus, people could understand and distinguish between links and the main content.

**Content availability and Readability:** Appropriate and updated content should be on the website to attract the target audience. Additionally, the readability of the content should be ensured.

**Redundant templates:** Sometimes, web developers consider the same template for multiple website development. This introduces barriers and increases the chances of frequently misusing several functions. Therefore, using the same template may prove to be a poor decision; developers should adapt themselves with new templates and updated technology.

**ARIA (Accessible Rich Internet Applications) attributes:** Around 50% of the websites did not use the ARIA function. To increase accessibility, web developers should use ARIA attributes in the HTML script.

## 8. Conclusions and Future Work

This paper presents an accessibility analysis of official government COVID-19 vaccine information websites for different countries of Europe and Asia. To assess the accessibility of the target websites, we employed four open access automatic web accessibility tools and human observation techniques. The evaluation results and the human judgment indicated that several accessibility issues existed in those targeted websites. Through both automatic evaluation and human observation, we identified the most frequent problems of the websites. We compared European and Asian countries’ results and found few differences in their accessibility. Based on the evaluation, this paper added some suggestions to improve the accessibility of these targeted websites. Through the findings of this paper, we wish to emphasize that, if web developers provide their services or develop the websites without following accessibility guidelines, our research on its own will not be enough to provide an accessible solution. Another remark from the evaluation is that most websites were not tested by people with visual challenges. Besides, due to cost efficiency, developers often reuse the design/development template instead of using an updated version. Therefore, we think that the findings of this study might help web developers and web designers to understand the drawbacks of their developments. In total, we considered only 21 websites from 13 countries. Based on the results of this research, we will incorporate websites from other countries from these two continents and other automated and human testing systems to generate a more systematic result. We will consider all the economic and social issues in our future research (population, GDP, etc.) to perform a depth analysis of these two continents’ results regarding web accessibility. Other countries from other continents should be considered and included in similar research in the future. Additionally, this research and the experiments were performed based on the WCAG 2.0 and WCAG 2.1 guidelines. In the future, we will continue our research based on WCAG 2.2 and WCAG 3.0 guidelines.

## Figures and Tables

**Table 1:** Considered selective Government websites.

No.	W-ID	Country	Region	Page-URL
1	WID-1.0	Estonia	Europe	https://vaktsineeri.ee/en/ (accessed on 1 July 2021)
2	WID-2.0	France	Europe	https://chis.cern/covid-19-vaccination-residents-france (accessed on 1 July 2021)
3	WID-3.0	Greece	Europe	https://help.unhcr.org/greece/coronavirus/ (accessed on 1 July 2021)
4	WID-4.0	Ireland	Europe	https://www.hse.ie/eng/ (accessed on 1 July 2021)
5	WID-4.1	Ireland	Europe	https://www2.hse.ie/coronavirus/ (accessed on 1 July 2021)
6	WID-5.0	Latvia	Europe	https://covid19.gov.lv/index.php/en/vaccine (accessed on 1 July 2021)
7	WID-6.0	Luxembourg	Europe	https://covid19.public.lu/en/vaccination.html (accessed on 1 July 2021)
8	WID-7.0	Netherland	Europe	https://coronadashboard.government.nl/landelijk/vaccinaties (accessed on 1 July 2021)
9	WID-8.0	Iceland	Europe	https://www.covid.is/statistical-information-on-vaccination (accessed on 1 July 2021)
10	WID-9.0	Switzerland	Europe	https://www.ch.ch/de/coronavirus/ (accessed on 1 July 2021)
11	WID-10.0	Croatia	Europe	https://www.koronavirus.hr/en (accessed on 1 July 2021)
12	WID-11.0	Germany	Europe	https://www.bundesgesundheitsministerium.de/en/topics/vaccinations.html (accessed on 1 July 2021)
13	WID-12.0	Austria	Europe	https://www.sozialministerium.at/ (accessed on 1 July 2021)
14	WID-13.0	India	Asia	https://www.mohfw.gov.in/ (accessed on 1 July 2021)
15	WID-14.0	Philippines	Asia	https://doh.gov.ph/ (accessed on 1 July 2021)
16	WID-15.0	Jordan	Asia	https://corona.moh.gov.jo/en (accessed on 1 July 2021)
17	WID-16.0	UAE	Asia	https://covid19.ncema.gov.ae/en (accessed on 1 July 2021)
18	WID-17.0	Kyrgyzstan	Asia	http://med.gov.kg/en/informatsii-2.html (accessed on 1 July 2021)
19	WID-18.0	Maldives	Asia	https://covid19.health.gov.mv/en/?c=0 (accessed on 1 July 2021)
20	WID-19.0	Hong Kong	Asia	https://www.covidvaccine.gov.hk/en/ (accessed on 1 July 2021)
21	WID-20.0	Qatar	Asia	https://www.gco.gov.qa/en/focus/covid-19/ (accessed on 1 July 2021)

**Table 2:** System usability testing questionnaire with evaluation scale.

Questions	Scale
1. I think that I would like to use this website frequently	1/2/3/4/5
2. I found this website unnecessarily complex.	1/2/3/4/5
3. I thought this website was easy to use.	1/2/3/4/5
4. I think that I would need assistance to be able to use this website.	1/2/3/4/5
5. I found the various functions in this website were well integrated.	1/2/3/4/5
6. I thought there was too much inconsistency in this website.	1/2/3/4/5
7. I would imagine that most people would learn to use this website very quickly	1/2/3/4/5
8. I found this website very cumbersome/awkward to use.	1/2/3/4/5
9. I felt very confident using this website.	1/2/3/4/5
10. I needed to learn a lot of things before I could get going with this website.	1/2/3/4/5

**Table 3:** System Usability testing by volunteers.

Information	Group-1						Group-2			
No of students	St-1	St-2	St-3	St-4	St-5	St-6	St-1	St-2	St-3	St-4
No of websites	2	2	2	2	2	3	2	2	2	2
Total	Students = 6, Websites = 13						Students = 4, Websites = 8			
Remarks	St = student Total participate students = 10, Total evaluated websites = 21									

**Table 4:** Expert testing questionnaire with evaluation scale.

Questions	Scale
1. Is the website accessible for a blind people/low vision people?	Yes/No
2. Is the website accessible for a person with a moving disability?	Yes/No
3. Is the link accessible or available?	Yes/No
4. Does the website information have color deficiency?	Yes/No
5. Does the website require CAPTCHA?	Yes/No
6. Is the website accessible through a keyboard?	Yes/No
7. Is the website an English version website?	Yes/No
8. Does the website have native language version?	Yes/No
9. Is the website responsive?	Yes/No
10. Does the website have inaccessible features?	Yes/No
11. Are links and buttons separable?	Yes/No
12. Does it allow manual font size adjustment?	Yes/No

**Table 5:** Mauve test: Government website accessibility testing result for WCAG 2.0 and WCAG 2.1.

Website Info		WCAG 2.0 (Conformance Level-AAA)						WCAG 2.1 (Conformance Level-AAA)					
Country	W-ID	P	O	U	R	Errors	Performance	P	O	U	R	Errors	Performance
EU	WID-1.0	3	1	1	0	5 (0.05)	High	9	1	1	0	11 (0.011)	Low
EU	WID-2.0	3	1	2	1	7 (0.07)	Medium	7	1	1	2	11 (0.011)	Low
EU	WID-3.0	3	1	2	1	7 (0.07)	Medium	8	1	1	1	11 (0.011)	Low
EU	WID-4.0	3	2	1	1	7 (0.07)	Medium	5		0	1	6 (0.06)	Medium
EU	WID-4.1	4	3	0	0	7 (0.07)	Medium	10	2	0	1	13 (0.013)	Low
EU	WID-5.0	4	1	2	1	8 (0.08)	Medium	12	1	0	1	14 (0.014)	Low
EU	WID-6.0	4	1	0	0	5 (0.05)	High	9	0	0	0	9 (0.09)	Medium
EU	WID-7.0	5	0	1	2	8 (0.08)	Medium	11	0	0	1	12 (0.012)	Low
EU	WID-8.0	2	1	0	1	4 (0.04)	High	3	1	0	0	4 (0.04)	High
EU	WID-9.0	3	0	0	0	3 (0.03)	High	7	0	0	0	7 (0.07)	Medium
EU	WID-10.0	6	2	1	2	11 (0.011)	Low	13	1	2	1	17 (0.017)	Low
EU	WID-11.0	8	1	3	0	12 (0.012)	Low	13	1	2	0	16 (0.016)	Low
EU	WID-12.0	-	-	-	-	-	-	-	-	-	-	-	-
Asia	WID-13.0	8	1	2	1	12 (0.012)	Low	10	2	1	0	13 (0.013)	Low
Asia	WID-14.0	9	3	1	1	14 (0.014)	Low	8	2	1	3	14 (0.014)	Low
Asia	WID-15.0	6	2	2	1	11 (0.011)	Low	6	1	2	1	10 (0.010)	Low
Asia	WID-16.0	3	1	1	1	6 (0.06)	Medium	5	1	2	1	9 (0.09)	Medium
Asia	WID-17.0	8	2	2	1	13 (0.013)	Low	8	2	1	1	12 (0.012)	Low
Asia	WID-18.0	7	2	1	1	11 (0.011)	Low	7	1	1	2	11 (0.011)	Low
Asia	WID-19.0	0	0	0	0	0 (0.0)	High	0	0	0	0	0 (0.0)	High
Asia	WID-20.0	2	0	2	2	6 (0.06)	Medium	3	0	1	2	6 (0.06)	Medium

**Table 6:** Nibbler test result for twenty-eight government COVID-19 information websites.

W-ID	Overall Score	Errors	Accessibility Score	Experience	Marketing	Technology	Performance
WID-1.0	9.10	8	9.4	8.6	7.5	9.6	Medium
WID-2.0	8.00	13	9.8	7.7	5.7	8.4	Low
WID-3.0	9.0	9	9.7	8.0	6.3	9.5	Medium
WID-4.0	8.70	19	9.6	8.8	9.4	8.0	Low
WID-4.1	8.70	19	9.6	8.8	9.4	8.0	Low
WID-5.0	8.70	12	9.7	8.1	7.1	8.4	Low
WID-6.0	6.90	20	7.3	5.1	5.5	7.1	Low
WID-7.0	8.60	10	9.7	7.4	5.8	9.0	Low
WID-8.0	8.90	13	9.4	8.1	7.4	9.2	Low
WID-9.0	8.70	16	9.9	9.3	8.5	9.2	Low
WID-10.0	9.0	15	7.8	8.1	9.7	8.7	Medium
WID-11.0	9.70	11	9.6	9.8	10	9.6	High
WID-12.0	8.50	18	9.0	9.2	8.7	9.2	Low
WID-13.0	8.60	26	8.5	9.4	9.2	8.5	Low
WID-14.0	8.70	17	9.1	9.6	8.8	8.6	Low
WID-15.0	7.80	15	7.5	7.6	6.4	8.0	Low
WID-16.0	8.50	17	9.5	8.2	6.7	8.2	Low
WID-17.0	8.0	23	8.6	8.1	6.8	7.9	Low
WID-18.0	7.90	19	8.9	7.1	5.6	8.1	Low
WID-19.0	8.10	11	8.3	8.1	6.0	8.6	Low
WID-20.0	8.90	19	9.6	8.7	9.2	8.3	Low

**Table 7:** Accessibility testing result for WAVE tool.

W-ID	Features	Errors	Contrast Errors	Alerts	Structural Elements	ARIA	Performance
WID-1.0	16	0	5	16	37	120	P
WID-2.0	3	3	3	16	25	108	F
WID-3.0	2	4	8	31	52	39	F
WID-4.0	4	3	3	2	22	16	F
WID-4.1	15	6	0	14	43	44	F
WID-5.0	48	0	2	6	36	18	P
WID-6.0	34	2	1	19	44	31	F
WID-7.0	16	4	27	14	41	49	F
WID-8.0	9	1	0	4	7	3	F
WID-9.0	23	4	0	31	20	3	F
WID-10.0	4	4	0	20	66	32	F
WID-11.0	16	31	16	19	35	29	F
WID-12.0	78	6	0	189	244	318	F
WID-13.0	29	29	15	473	52	26	F
WID-14.0	19	25	100	63	39	12	F
WID-15.0	8	59	39	5	42	2	F
WID-16.0	1	10	12	43	11	0	F
WID-17.0	35	11	22	33	30	28	F
WID-18.0	40	47	25	33	24	32	F
WID-19.0	1	0	0	3	0	0	P
WID-20.0	8	2	6	4	36	0	F

**Table 8:** WEB accessibility tool result considering number of violations.

W-ID	Compliance Score	No of Violations	Performance
WID-1.0	84%	17	F
WID-2.0	92%	0	P
WID-3.0	86%	6	F
WID-4.0	87%	1	F
WID-4.1	81%	5	F
WID-5.0	88%	1	F
WID-6.0	83%	5	F
WID-7.0	83%	104	F
WID-8.0	85%	3	F
WID-9.0	86%	9	F
WID-10.0	86%	18	F
WID-11.0	90%	1	F
WID-12.0	92%	0	P
WID-13.0	75%	8	F
WID-14.0	78%	26	F
WID-15.0	80%	23	F
WID-16.0	77%	11	F
WID-17.0	78%	24	F
WID-18.0	76%	66	F
WID-19.0	85%	2	F
WID-20.0	90%	1	F

**Table 9:** SUS result of targeted websites.

W-ID	Country	SUS Score	Remarks
WID-1.0	Estonia	75.0	Good
WID-2.0	France	97.5	Best
WID-3.0	Greece	100	Best
WID-4.0	Ireland	40.0	Poor
WID-4.1	Ireland	25.0	Worst
WID-5.0	Latvia	72.5	Good
WID-6.0	Luxembourg	72.5	Good
WID-7.0	Netherland	75.0	Good
WID-8.0	Iceland	65.0	Ok/Fair
WID-9.0	Schweiz	42.5	Poor
WID-10.0	Croatia	57.5	Ok/Fair
WID-11.0	Germany	65.0	Ok/Fair
WID-12.0	Austria	82.5	Excellent
WID-13.0	India	50.0	Ok/Fair
WID-14.0	Philippines	70.0	Good
WID-15.0	Jordan	85.0	Excellent
WID-16.0	UAE	20.0	Worst
WID-17.0	Kyrgyzstan	35.0	Poor
WID-18.0	Maldives	35.0	Poor
WID-19.0	Hong Kong	80.0	Excellent
WID-20.0	Qatar	75.0	Good

**Table 10:** Quality assessment parameter.

SUS Score	Quality Assessment
90–100	Best
80–89	Excellent
70–79	Good
50–69	Ok/Fair
30–49	Poor
1–29	Worst

**Table 11:** Expert result of targeted websites.

Questions	Expert-1		Expert-2		Expert-3		Expert-4	
	Yes	No	Yes	No	Yes	No	Yes	No
1. Is the website accessible for a blind people/low vision people?	7	14	4	17	9	12	6	15
2. Is the website accessible for a person with a moving disability?	18	3	12	9	9	12	20	1
3. Is the link accessible or available?	21	0	20	1	15	6	18	3
4. Does the website information have color deficiency?	13	8	20	1	15	6	10	11
5. Does the website require CAPTCHA?	1	20	0	21	1	20	3	18
6. Is the website accessible through a keyboard?	20	1	15	6	21	0	18	3
7. Is the website an English version website?	14	4	10	11	12	9	14	7
8. Does the website have native language version?	15	6	17	4	16	5	14	7
9. Is the website responsive?	16	5	20	1	15	6	14	7
10. Does the website have inaccessible features?	4	17	8	13	10	11	5	16
11. Are links and buttons separable?	13	8	10	11	12	9	15	6
12. Does it allow manual font size adjustment?	0	21	0	21	0	21	0	21

**Table 12:** Average number of errors of each testing tools.

Testing Tools	Europe	Asia
Mauve++	9.5	9.0
Nibbler	14.0	18.0
WAVE	5.0	22.0
Web accessibility	13.0	20.0
**Total Average number of errors**	**10.37**	**17.25**

**Table 13:** Questionnaire-based System Usability Score.

Country	Questions										
	Q1	Q2	Q3	Q4	Q5	Q6	Q7	Q8	Q9	Q10	Total
Estonia	4	4	0	4	2	4	1	4	3	4	75
France	4	4	4	4	4	4	4	4	3	4	97.5
Greece	4	4	4	4	4	4	4	4	4	4	100.0
Ireland-1	0	3	0	1	0	4	0	4	0	4	40.0
Ireland-2	1	0	0	0	1	4	1	2	0	1	25.0
Latvia	1	4	4	4	1	4	2	4	2	3	72.5
Luxembourg	1	4	4	4	1	4	2	4	2	3	72.5
Netherland	4	3	2	3	3	3	2	4	3	3	75.0
Iceland	2	3	1	3	2	4	2	3	3	3	65.0
Schweiz	0	4	1	1	0	4	1	3	0	3	42.5
Croatia	2	2	3	4	2	1	1	3	2	3	57.5
Germany	2	3	3	4	2	3	1	3	2	3	65.0
Austria	4	3	3	4	4	3	3	3	3	3	82.5
**Average SUS Score**											**66.92**
India	1	3	2	3	1	3	1	3	0	3	50.0
Philippines	2	3	4	4	2	3	2	4	1	3	70.0
Jordan	4	3	4	4	2	4	3	4	2	4	85.0
UAE	0	0	2	2	1	2	0	0	0	1	20.0
Kyrgyzstan	0	2	1	4	0	0	2	1	0	4	35.0
Maldives	0	2	1	4	0	0	2	1	0	4	35.0
Hong Kong	3	4	4	3	3	3	2	4	2	4	80.0
Qatar	1	4	4	4	1	3	4	4	1	4	75.0
**Average SUS Score**											**56.25**

**Table 14:** Expert questionnaire-based websites requirements and accessibility score.

Country	Questions												
	Blind Version	Moving Disability	Link Purpose	Color Deficiency	CAPTCHA	Keyboard Access	English Version	Native Version	Responsive	Inaccessible Feature	Link/ Button Visibility	Font Size	Total
Estonia	0	1	1	1	1	1	1	1	1	1	0	0	0.75
France	1	0	1	1	1	1	1	1	1	1	1	0	0.83
Greece	0	1	1	0	1	1	1	1	1	1	1	0	0.75
Ireland-1	0	1	1	0	1	1	1	0	1	0	1	0	0.58
Ireland-2	0	0	1	0	1	1	1	0	1	0	1	0	0.50
Latvia	1	0	1	1	1	1	1	1	1	1	0	0	0.75
Luxembourg	0	1	1	0	1	1	1	1	1	1	1	0	0.75
Netherland	1	1	1	0	1	1	1	1	0	1	0	0	0.66
Iceland	1	1	1	1	1	1	1	1	1	1	0	0	0.83
Schweiz	0	1	1	0	1	1	0	1	0	1	1	0	0.58
Croatia	1	1	1	1	1	1	1	0	1	0	1	0	0.75
Germany	0	1	1	0	1	1	1	0	1	0	0	0	0.50
Austria	0	1	1	0	1	1	1	1	1	1	0	0	0.66
**Average** **Score**	**0.41**	**0.76**	**1.0**	**0.38**	**1.0**	**1.0**	**0.92**	**0.69**	**0.84**	**0.69**	**0.53**	**0.0**	**0.68**
India	0	1	1	0	0	1	1	0	1	1	0	0	0.50
Philippines	1	1	1	1	1	1	1	0	1	1	1	0	0.83
Jordan	0	1	1	0	1	1	1	1	0	1	1	0	0.66
UAE	1	1	1	1	1	1	1	1	1	0	1	0	0.83
Kyrgyzstan	0	1	1	0	1	1	1	1	1	1	1	0	0.75
Maldives	0	0	1	0	1	0	1	1	0	1	1	0	0.50
Hong Kong	1	1	1	1	1	1	1	1	1	1	1	0	0.91
Qatar	1	1	1	1	1	1	1	1	0	1	0	0	0.75
**Average** **Score**	**0.5**	**0.12**	**1.0**	**0.5**	**0.87**	**0.87**	**1.0**	**0.75**	**0.62**	**0.87**	**0.75**	**0.0**	**0.71**

**Table 15:** Frequently appeared errors with their associated principles.

Error Type	Principles	No. of Errors
1.1.1, 1.3.1, 1.3.4, 1.3.5, 1.3.6, 1.4.1, 1.4.4, 1.4.5, 1.4.6, 1.4.8, 1.4.9, 1.4.10, 1.4.11, 1.4.12, 1.4.13	Perceivable	285
2.2.2, 2.2.4, 2.4.1, 2.4.2, 2.4.4, 2.4.6, 2.4.7, 2.4.9, 2.4.10	Operable	124
3.1, 3.1.1, 3.1.2, 3.1.5, 3.2.1, 3.2.2, 3.2.4, 3.2.5, 3.3.1, 3.3.2, 3.3.3, 3.3.4,	Understandable	144
4.1.1, 4.1.2	Robust	52

## Data Availability

Data are available on request.

## Abbreviations

The following abbreviations are used in this manuscript:

WCAG	Websites Content Accessibility Guidelines
WAI	Web Accessibility Initiative
WWW	World Wide Web
WHO	World Health Organization
SUS	System Usability Scale
JSON	JavaScript Object Notation
HTML	Hypertext Markup Language
CSS	Cascading Style Sheets
ARIA	Accessible Rich Internet Application
P	Perceivable
O	Operable
U	Understandable
R	Robust
C	Conformance

## Appendix A

**Table A1:** Accessibility issues in the website of each country for WCAG 2.0.

Website Region	Mauve++ Tool (WCAG-2.0)		
	Violations	Success Criteria	Principles
Estonia	Headings	1.3.1: Info and relationships	P
	Alignment in CSS	1.4.10: Reflow	P
	Line space in CSS	1.4.10: Reflow	P
	Styling element	2.4.7: Focus Visible	O
	Language attribute	3.1.1: Language of page	U
France	Line space/Alignment in CSS	1.4.8: Visual Presentation	P
	Font size	1.4.4: Resize text	P
	Label element	1.1.1: Non text context	P
	Styling element outlines and borders	2.4.7: Focus Visible	O
	Title attribute	3.3.2: Labels or instructions	U
	Button	3.3.2: Labels or instructions	U
	Title attribute	4.1.2: Name, Role, Value	R
Greece	Font size	1.4.4: Resize text	P
	Headings	1.3.1: Info and relationships	P
	Line space/Alignment in CSS	1.4.8: Visual Presentation	P
	Frame and iframe elements	2.4.1: Bypass Blocks	O
	Form controls for label element	3.3.2: Labels or instructions	U
	Language attributes	3.1.1: Language of Page	U
	Title attribute of the frame and iframe elements	4.1.2: Name, Role, Value	R
Ireland-1	Line spacing in CSS	1.4.8: Visual Presentation	P
	Headings	1.3.1: Info and relationships	P
	Label element	1.1.1: Non text context	P
	Styling element outlines and borders	2.4.7: Focus Visible	O
	Frame and iframe elements	2.4.1: Bypass Blocks	O
	Title attribute	3.3.2: Labels or instructions	U
	Form controls	4.1.2: Name, Role, Value	R
Ireland-2	Line space/Alignment in CSS	1.4.8: Visual Presentation	P
	Headings	1.3.1: Info and relationships	P
	Label element	1.1.1: Non text context	P
	Percent, em units or named font sizes	1.4.4: Resize text	P
	Frame and iframe elements	2.4.1: Bypass Blocks	O
	Styling element outlines and borders	2.4.7: Focus Visible	O
	Title attribute	2.4.1: Bypass Blocks	O
Latvia	Line space/Alignment in CSS	1.4.8: Visual Presentation	P
	Headings	1.3.1: Info and relationships	P
	Contrast ratio	1.4.6: Contrast (Enhanced)	P
	Styling element outlines and borders	2.4.7: Focus Visible	O
	Title attribute	3.3.2: Labels or instructions	U
	Label element	4.1.2: Name, Role, Value	R
	Percent, em units or named font sizes	1.4.4: Resize text	P
Luxembourg	Line spacing in CSS	1.4.8: Visual Presentation	P
	Alignment	1.4.8: Visual Presentation	P
	Percent, em units or named font sizes	1.4.4: Resize text	P
	Contrast ratio	1.4.6: Contrast (Enhanced)	P
	Styling element outlines and borders	2.4.7: Focus Visible	O
Netherland	Line space/Alignment in CSS	1.4.8: Visual Presentation	P
	Fieldset and legend elements	1.3.1: Info and relationships	P
	Header cells and data cells	1.3.1: Info and relationships	P
	Alt attribute on img elements	1.1.1: Non text context	P
	Label element	1.1.1: Non text context	P
	Title attribute of form controls	3.3.2: Labels or instructions	U
	Aria-labell	4.1.2: Name, Role, Value	R
	Interface component	4.1.2: Name, Role, Value	R
Iceland	Link text	1.1.1: Non text context	P
	Headings	1.3.1: Info and relationships	P
	Title attribute of the frame and iframe elements	2.4.1: Bypass Blocks	O
	Role attribute	4.1.2: Name, Role, Value	R
Switzerland	Line spacing in CSS	1.4.8: Visual Presentation	P
	Percent, em units or named font sizes	1.4.4: Resize text	P
	Alignment in CSS	1.4.8: Visual Presentation	P
Croatia	Line space/Alignment in CSS	1.4.8: Visual Presentation	P
	Percent, em units or named font sizes	1.4.4: Resize text	P
	Title attribute	1.1.1: Non text context	P
	Information and structure	1.4.9: Images of Text (No Exception)	P
	Headings	1.3.1: Info and relationships	P
	Label elements to associate text	1.3.1: Info and relationships	P
	Styling element outlines and borders	2.4.7: Focus Visible	O
	Submit buttons	2.2.2: Pause, Stop, Hide	O
	Language attributes	3.1.1: Language of Page	U
	Id attributes	4.1.1: Non text context	R
	Label elements to associate text	4.1.2: Name, Role, Value	R
Germany	Line space/Alignment in CSS	1.4.8: Visual Presentation	P
	Link text	1.1.1: Non text context	P
	Percent, em units or named font sizes	1.4.5: Images of Text	P
	Title attribute	1.3.1: Info and relationships	P
	Information and structure	1.4.9: Images of Text (No Exception)	P
	Headings	1.3.1: Info and relationships	P
	Label elements	1.1.1: Non text context	P
	Contrast ratio	1.4.6: Contrast (Enhanced)	P
	Styling element outlines and borders	2.4.7: Focus Visible	O
	Title attribute of form controls	3.3.2: Labels or instructions	U
	Submit buttons	3.2.2: On Input	U
	Language attributes	3.1.1: Language of Page	U
Austria	-	-	-
India	Line space/Alignment in CSS	1.4.8: Visual Presentation	P
	Link text	1.1.1: Non text context	P
	Alt attribute on img elements, area elements, and input elements	1.1.1: Non text context	P
	Header cells and data cells	1.3.1: Info and relationships	P
	Percent, em units or named font sizes	1.4.4: Resize text	P
	Title attribute	1.3.1: Info and Relationships	P
	Information and structure	1.4.9: Images of Text (No Exception)	P
	Contrast ratio	1.4.6: Contrast (Enhanced)	P
	Styling element outlines and borders	2.4.7: Focus Visible	O
	Title attribute to identify form controls	3.3.2: Labels or instructions	U
	Language attributes	3.1.1: Language of Page	U
	Label element	4.1.2: Name, Role, Value	R
Philippines	Line space/Alignment in CSS	1.4.8: Visual Presentation	P
	Link text	1.1.1: Non text context	P
	Alt attribute on img elements, area elements, and input elements	1.1.1: Non text context	P
	Percent, em units or named font sizes	1.4.5: Images of Text	P
	Title attribute to identify form controls	1.3.1: Info and relationships	P
	Headings	1.3.1: Info and relationships	P
	Information and structure	1.4.9: Images of Text (No Exception)	P
	Label elements	1.3.1: Info and relationships	P
	Contrast ratio	1.4.6: Contrast (Enhanced)	P
	Link text	2.4.4: Link Purpose (In Context)	O
	Styling element outlines and borders	2.4.7: Focus Visible	O
	Title attribute of the frame and iframe elements	2.4.1: Bypass Blocks	O
	Title attribute to identify form controls	3.3.2: Labels or Instructions	U
	Label elements to associate text labels	4.1.2: Name, Role, Value	R
Jordan	Link text	1.1.1: Non text context	P
	Alt attribute on img elements, area elements, and input elements	1.1.1: Non text context	P
	Line space/Alignment in CSS	1.4.8: Visual Presentation	P
	Percent, em units or named font sizes	1.4.4: Resize text	P
	Headings	1.3.1: Info and relationships	P
	Contrast ratio	1.4.6: Contrast (Enhanced)	P
	Title attribute of the frame and iframe elements	2.4.1: Bypass Blocks	O
	Submit buttons	3.2.2: On Input	U
	Language attributes	3.1.1: Language of Page	U
	Title attribute of the frame and iframe elements	4.1.2: Name, Role, Value	R
	Anchor elements	2.4.4: Link Purpose (In Context)	O
UAE	Alt attribute on img elements, area elements, and input elements	1.1.1: Non text context	P
	Information and structure	1.3.1: Info and relationships	P
	Label elements to associate text	1.1.1: Non text context	P
	Link text	2.4.4: Link Purpose (In Context)	O
	Submit buttons	3.2.2: On Input	U
	Label elements to associate text	4.1.2: Name, Role, Value	R
Kyrgyzstan	Line space/Alignment in CSS	1.4.8: Visual Presentation	P
	Link text	1.1.1: Non text context	P
	Alt attribute on img elements, area elements, and input elements	1.1.1: Non text context	P
	Percent, em units or named font sizes	1.4.4: Resize text	P
	Title attribute	1.3.1: Info and relationships	P
	Information and structure	1.4.9: Images of Text (No Exception)	P
	Headings	1.3.1: Info and relationships	P
	Contrast ratio	1.4.6: Contrast (Enhanced)	P
	Anchor elements	2.2.4: Interruptions	O
	Styling element outlines and borders	2.4.7: Focus Visible	O
	Submit buttons	3.2.2: On Input	U
	Language attributes	3.1.1: Language of Page	U
	Title attribute of form controls	4.1.2: Name, Role, Value	R
Maldives	Column widths	1.4.4: Resize text	P
	Link text	1.1.1: Non text context	P
	Line space/Alignment in CSS	1.4.8: Visual Presentation	P
	Percent, em units or named font sizes	1.4.9: Images of Text (No Exception)	P
	Information and structure	1.3.1: Info and relationships	P
	Headings	1.3.1: Info and relationships	P
	Contrast ratio	1.4.6: Contrast (Enhanced)	P
	Link for anchor elements	2.4.4: Link Purpose (In Context)	O
	Title attribute of the frame and iframe elements	2.4.1: Bypass Blocks	O
	Language attributes	3.1.1: Language of Page	U
	Title attribute of the frame and iframe elements	4.1.2: Name, Role, Value	R
Hong Kong	-	-	-
Qatar	Headings	1.3.1: Info and relationships	P
	Label elements	1.1.1: Non text context	P
	Title attribute	3.3.2: Labels or instructions	U
	Language attributes	3.1.1: Language of Page	U
	Id attributes	4.1.1: Parsing	R
	Label elements	4.1.2: Name, Role, Value	R

**Table A2:** Accessibility issues in the website of each country for WCAG 2.1.

Website Region	Mauve++ Tool (WCAG 2.1)		
	Violations	Success Criteria	Principles
Estonia	Size and position	1.4.10: Reflow	P
	Internal links	1.4.1: Use of color	P
	landscape or portrait view	1.3.4: Orientation	P
	Input	1.3.5: Identify Input Purpose	P
	Alignment in CSS	1.4.8: Visual Presentation	P
	Line space in CSS	1.4.10: Reflow	P
	Focus indicator	1.4.11: Non text contrast	P
	ARIA landmark	1.3.6: Identify Purpose	P
	Headings	1.3.1: Info and relationships	P
	Styling element	2.4.7: Focus Visible	O
	Language attribute	3.1.1: Error Identification	U
France	Size and position	1.4.10: Reflow	P
	Internal links	1.4.1: Use of color	P
	Line or alignment in CSS	1.4.10: Reflow	P
	Title attribute to identify form controls	1.3.1: Info and relationships	P
	ARIA landmarks	1.3.6: Identify Purpose	P
	Percent, em units or named font sizes	1.4.4: Resize text	P
	Alignment	1.4.8: Visual Presentation	P
	Styling element outlines and borders	2.4.7: Focus Visible	O
	Submit buttons	3.2.2: On Input	U
	Title attribute to identify form controls	4.1.1: Parsing	R
	Aria-labelled	4.1.2: Name, Role, Value	R
Greece	Title attribute	1.1.1: Non text content	P
	Headings	1.3.1: Info and relationships	P
	Size and position	1.4.10: Reflow	P
	Information and structure	1.4.5: Images of Text	P
	ARIA landmarks	1.3.6: Identify Purpose	P
	CSS width, max-width and flexbox	1.4.10: Reflow	P
	Line space or alignment in CSS	1.4.8: Visual Presentation	P
	Percent, em units or named font sizes	1.4.4: Resize text	P
	Title attribute of the frame and iframe elements	2.4.1: Bypass Blocks	O
	Language attributes	3.1.1: Language of Page	U
	Title attribute of the frame and iframe elements	4.1.2: Name, Role, Value	R
Ireland-1	Size and position	1.4.10: Reflow	P
	Internal links	1.4.1: Use of color	P
	Styling element	1.4.11: Non text contrast	P
	Headings	1.3.1: Info and relationships	P
	Percent, em units or named font sizes	1.4.4: Resize text	P
	ARIA landmarks	4.1.2: Name, role, value	R
Ireland-2	Size and position	1.4.10: Reflow	P
	Hover and focus pseudo classes	1.4.13: Content on Hover or Focus	P
	Title attribute of form controls	1.1.1: Non text content	P
	Styling element outlines and borders	1.4.11: Non-text Contrast	P
	Headings	1.3.1: Info and Relationships	P
	ARIA landmarks	1.3.6: Identify Purpose	P
	CSS width, max-width and flexbox	1.4.10: Reflow	P
	Alignment in CSS	1.4.8: Visual Presentation	P
	Percent, em units or named font sizes	1.4.4: Resize text	P
	Contrast ratio	1.4.11: Non-text Contrast	P
	Styling element outlines and borders	2.4.7: Focus Visible	O
	Title attribute of the frame and iframe elements	2.4.1: Bypass Blocks	O
	Label element	4.1.2: Name, Role, Value	R
Latvia	Hover and focus	1.4.13: Content on Hover or Focus	P
	Title attribute of form controls	1.1.1: Non text content	P
	Styling element outlines and borders	1.4.11: Non text contrast	P
	Links	1.4.1: Use of Color	P
	Purpose of inputs	1.3.5: Identify Input Purpose	P
	Focus indicator	1.4.11: Non text contrast	P
	Headings	1.3.1: Info and relationships	P
	Alignment in CSS	1.4.8: Visual presentation	P
	Percent, em units or named font sizes	1.4.4: Resize text	P
	Contrast ratio	1.4.6: Contrast enhanced	P
	Size and position	1.4.10: Reflow	P
	ARIA landmarks	1.3.6: Identify purpose	P
	Focus Indicator	2.4.7: Focus Visible	O
	Label element	4.1.1: Parsing	R
Luxembourg	Focus indicator	1.4.11: Non text contrast	P
	Inputs	1.3.5: Identify Input Purpose	P
	Size and position	1.4.10: Reflow	P
	Internal links	1.4.1: Use of color	P
	Styling element	1.4.11: Non text contrast	P
	Alignment	1.4.8: Visual presentation	P
	Contrast ratio	1.4.6: Contrast (Enhanced)	P
	CSS width, max-width and flexbox	1.4.10: Reflow	P
	Contrast ratio	1.4.6: Contrast (Enhanced)	P
Netherland	Size and position	1.4.10: Reflow	P
	Internal links	1.4.1: Use of color	P
	Field set and legend elements	1.3.1: Info and relationships	P
	Header cells and data cells	1.3.1: Info and relationships	P
	Hover and focus pseudo classes	1.4.13: Content on Hover or focus	P
	Headings	1.3.1: Info and relationships	P
	Alignment	1.4.8: Visual presentation	P
	Alt attribute on img elements, area elements, and input elements	1.1.1: Non-text Content	P
	Label element	1.1.1: Non-text Content	P
	Focus indicator	1.4.11: Non-text Contrast	P
	Contrast ratio	1.4.6: Contrast (Enhanced)	P
	Aria-labelled	4.1.2: Name, Role, Value	R
Iceland	Link text	1.1.1: Non text content	P
	Headings	1.3.1: Info and relationships	P
	ARIA landmarks	1.3.6: Identify purpose	P
	Title attribute of the frame and iframe elements	2.4.1: Bypass Blocks	O
Switzerland	Size and position	1.4.10: Reflow	P
	ARIA landmarks	1.3.6: Identify purpose	P
	Alignment	1.4.8: Visual presentation	P
	Percent, em units or named font sizes	1.4.4: Resize text	P
	CSS width, max-width and flexbox	1.4.10: Reflow	P
	Links	1.4.1: Use of Color	P
	Line spacing in CSS	1.4.12: Text Spacing	P
Croatia	Internal links	1.4.1: Use of color	P
	Styling element outlines and borders	1.4.11: Non text contrast	P
	Title attribute	1.3.1: Info and relationships	P
	Information and structure	1.4.5: Images of text	P
	Focus indicator	1.4.11: Non text contrast	P
	Headings	1.3.1: Info and relationships	P
	Alignment in CSS	1.4.8: Visual presentation	P
	Percent, em units or named font sizes	1.4.4: Resize text	P
	Size and position	1.4.10: Reflow	P
	ARIA landmarks	1.3.6: Identify purpose	P
	Labels and inputs	1.4.10: Reflow	P
	Label elements	1.1.1: Non-text Content	P
	Contrast ratio	1.4.11: Non text contrast	P
	Styling element outlines and borders	2.4.7: Focus Visible	O
	Submit buttons	3.2.2: On Input	U
	Language attributes	3.1.1: Language of Page	U
	Id attributes	4.1.1: Parsing	R
Germany	Internal links	1.4.1: Use of color	P
	Link text	1.1.1: Non text content	P
	Styling element outlines and borders	1.4.11: Non text contrast	P
	Title attribute	1.3.1: Info and relationships	P
	Inputs	1.3.5: Identify input purpose	P
	Information and structure	1.4.5: Images of text	P
	Focus indicator	1.4.11: Non text contrast	P
	Headings	1.3.1: Info and relationships	P
	Alignment in CSS	1.4.8: Visual presentation	P
	Percent, em units or named font sizes	1.4.5: Images of text	P
	Contrast ratio	1.4.6: Contrast (Enhanced)	P
	Size and position	1.4.10: Reflow	P
	ARIA landmarks	1.3.6: Identify purpose	P
	Anchor elements	2.4.4: Link Purpose (In Context)	O
	Submit buttons	3.2.2: On Input	U
	Language attributes	3.1.1: Language of Page	U
Austria	-	-	-
India	Size and position	1.4.10: Reflow	P
	Internal links	1.4.1: Use of colour	P
	Link text	1.1.1: Non text content	P
	Alt attribute on img elements, area elements, and input elements	1.1.1: Non text content	P
	Header cells and data cells	1.3.1: Info and relationships	P
	Hover and focus pseudo classes	1.4.13: Content on Hover or focus	P
	Styling element outlines and borders	1.4.11: Non text contrast	P
	Information and structure	1.3.1: Info and relationships	P
	Focus indicator	1.4.11: Non text contrast	P
	Headings	1.3.1: Info and relationships	P
	Anchor elements	2.4.4: Link Purpose (In Context)	O
	Styling element outlines and borders	2.4.7: Focus Visible	O
	Language attributes	3.1.1: Error Identification	U
Philippines	Size and position	1.4.10: Reflow	P
	Internal links	1.4.1: Use of colour	P
	Link text	1.1.1: Use of colour	P
	Alt attribute on img elements, area elements, and input elements	1.1.1: Use of colour	P
	Title attribute	1.1.1: Use of colour	P
	Headings	1.3.1: Info and relationships	P
	Information and structure	1.3.1: Info and relationships	P
	Percent, em units or named font sizes	1.4.4: Resize text	P
	Sstyling element outlines and borders	2.4.7: Focus Visible	O
	Title attribute of the frame and iframe elements	2.4.1: Bypass Blocks	O
	Label elements	3.3.2: Labels or Instructions	U
	Label elements	4.1.2: Name, Role, Value	R
	Aria-labelled	4.1.2: Name, Role, Value	R
	Title attribute to identify form controls	4.1.1: Parsing	R
Jordan	Size and position	1.4.10: Reflow	P
	Internal links	1.4.1: Use of colour	P
	Link text	1.1.1: Use of colour	P
	Alt attribute on img elements, area elements, and input elements	1.1.1: Use of colour	P
	Percent, em units or named font sizes	1.4.5: Image of text	P
	Headings	1.3.1: Info and relationships	P
	Link text	2.4.4: Link Purpose (In Context)	O
	Submit buttons	3.2.2: On Input	U
	Language attributes	3.1.1: Language of Page	U
	Title attribute of the frame and iframe elements	4.1.2: Name, Role, Value	R
UAE	Size and position	1.4.10: Use of colour	P
	Alt attribute on img elements, area elements, and input elements	1.1.1: Use of colour	P
	Information and structure	1.3.1: Info and relationships	P
	Headings	1.3.1: Info and relationships	P
	Alignment in CSS	1.1.1: Use of colour	P
	Link text	2.4.4: Link Purpose (In Context)	O
	Language attributes	3.1.1: Language of Page	U
	Submit buttons	3.2.2: On Input	U
	Label elements	4.1.2: Name, Role, Value	R
Kyrgyzstan	Size and position	1.4.10: Reflow	P
	Internal links	1.4.1: Use of colour	P
	Data tables	1.3.1: Info and relationships	P
	Alt attributeon img elements, area elements, and input elements	1.1.1: Use of colour	P
	Information and structure	1.3.1: Info and relationships	P
	Headings	1.3.1: Info and relationships	P
	Percent, em units or named font sizes	1.4.4: Resize text	P
	Alignment in CSS	1.4.10: Reflow	P
	Link text	2.4.4: Link Purpose (In Context)	O
	Styling element outlines and borders	2.4.7: Focus Visible	O
	Language attributes	3.1.1: Language of Page	U
	Title attribute of form controls	4.1.2: Name, Role, Value	R
Maldives	Column widths	1.4.4: Resize text	P
	Size and position	1.4.10: Reflow	P
	Internal links	1.4.1: Use of colour	P
	Anchor elements	1.1.1: Use of colour	P
	Headings	1.3.1: Info and relationships	P
	Percent, em units or named font sizes	1.4.4: Resize text	P
	Information and structure	1.4.5: Images of Text	P
	Link text	2.4.4: Link Purpose (In Context)	O
	Language attributes	3.1.1: Language of Page	U
	Title attribute of the frame and iframe elements	4.1.2: Name, Role, Value	R
	Aria-labelled	4.1.2: Name, Role, Value	R
Hong Kong	-	-	-
Qatar	Information and structure	1.3.1: Info and relationships	P
	Headings	1.3.1: Info and relationships	P
	Title attribute of form controls	1.1.1: Use of colour	P
	Language attributes	3.1.1: Language of Page	U
	Title attribute	4.1.1: Parsing	R
	Label elements	4.1.2: Name, Role, Value	R

**Table A3:** Accessibility issues in the website of each country by Nibbler.

Website Region	Nibbler Tool			
	Violations	No. of Occurrence	Successive Criteria	Principles
Estonia	Incoming and internal links	4	2.4.9 Link Purpose (Link Only)	O
	Popularity	2	3.2.4 Consistent Identification	U
	Amount of content	2	3.1. Readable	U
France	Analytics	1	3.3.1 Error Identification	U
			3.3.2 Labels or Instructions	U
			3.3.3 Error Suggestion	U
			3.3.4 Error Prevention (Legal, Financial, Data)	U
	Popularity	2	3.2.4 Consistent Identification	U
	Server Behaviour	2	3.2.5 Change on Request	U
	Meta tags	2	4.1.1 Parsing	R
	Amount of content	2	3.1. Readable	U
	Internal links	4	2.4.9 Link Purpose (Link Only)	O
Greece	Popularity	2	3.2.4 Consistent Identification	U
	Images	2	1.4.5 Images of Text	P
	Amount of content	2	3.1. Readable	U
	Mobile	3	5.2.4 Only Accessibility-Supported Ways of Using Technologies	C
Ireland-1	Freshness	2	2.2.2 Pause, Stop, Hide	O
	Images	2	1.4.5 Images of Text	P
	Meta tags	2	4.1.1 Parsing	R
	Popularity	2	3.2.4 Consistent Identification	U
	Headings	3	2.4.6 Headings and Labels	O
	Server behaviour	2	3.2.5 Change on Request	U
	Amount of content	2	3.1. Readable	U
	Internal links	4	2.4.9 Link Purpose (Link Only)	O
Ireland-2	Freshness	2	2.2.2 Pause, Stop, Hide	O
	Server behaviour	2	3.2.5 Change on Request	U
	Images	2	1.4.5 Images of Text	P
	Meta tags	2	4.1.1 Parsing	R
	Popularity	2	3.2.4 Consistent Identification	U
	Headings	3	2.4.6 Headings and Labels	O
	Internal links	4	2.4.9 Link Purpose (Link Only)	O
	Amount of content	2	3.1. Readable	U
Latvia	Meta tags	2	4.1.1 Parsing	R
	Server behaviour	2	3.2.5 Change on Request	U
	Popularity	2	3.2.4 Consistent Identification	U
	Internal links	4	2.4.9 Link Purpose (Link Only)	O
	Amount of content	2	3.1. Readable	U
Luxembourg	Analytics	1	3.3.1 Error Identification	U
			3.3.2 Labels or Instructions	U
			3.3.3 Error Suggestion	U
			3.3.4 Error Prevention (Legal, Financial, Data)	U
	Popularity	2	3.2.4 Consistent Identification	U
	Mobile	3	5.2.4 Only Accessibility-Supported Ways of Using Technologies	C
	URL format	3	4.1.2 Name, Role, Value	R
	Server behaviour	2	3.2.5 Change on Request	U
	Amount of content	2	3.1. Readable	U
	Internal link	4	2.4.9 Link Purpose (Link Only)	O
	Page title	2	2.4.2 Page Titled	O
	Images	1	1.4.5 Images of Text	P
Netherland	Printability	2	5.2.4 Only Accessibility-Supported Ways of Using Technologies	C
	Popularity	2	3.2.4 Consistent Identification	U
	Amount of content	2	3.1. Readable	U
	Server behaviour	2	3.2.5 Change on Request	U
	Images	2	1.4.5 Images of Text	P
Iceland	Printability	2	5.2.4 Only Accessibility-Supported Ways of Using Technologies	C
	Amount of content	2	3.1. Readable	U
	Popularity	2	3.2.4 Consistent Identification	U
	Headings	3	2.4.6 Headings and Labels	O
	Internal links	4	2.4.9 Link Purpose (Link Only)	O
Switzerland	Freshness	2	2.2.2 Pause, Stop, Hide	O
	Meta tags	2	4.1.1 Parsing	R
	Server behaviour	2	3.2.5 Change on Request	U
	Amount of content	2	3.1. Readable	U
	Popularity	2	3.2.4 Consistent Identification	U
	Internal links	4	2.4.9 Link Purpose (Link Only)	O
	Twitter	2	5.2.4 Only Accessibility-Supported Ways of Using Technologies	C
Croatia	Mobile	3	5.2.4 Only Accessibility-Supported Ways of Using Technologies	C
	Headings	3	2.4.6 Headings and Labels	O
	URL format	3	4.1.2 Name, Role, Value	R
	Amount of content	2	3.1. Readable	U
	Internal links	4	2.4.9 Link Purpose (Link Only)	O
Germany	Internal links	4	2.4.9 Link Purpose (Link Only)	O
	server behaviours	2	3.2.5 Change on Request	U
	Images	2	1.4.5 Images of Text	P
	Headings	3	2.4.6 Headings and Labels	O
Austria	Analytics	1	3.3.1 Error Identification	U
			3.3.2 Labels or Instructions	U
			3.3.3 Error Suggestion	U
			3.3.4 Error Prevention (Legal, Financial, Data)	U
	URL format	3	4.1.2 Name, Role, Value	R
	Meta tags	2	4.1.1 Parsing	R
	Popularity	2	3.2.4 Consistent Identification	U
	Amount of content	2	3.1. Readable	U
	Mobile	2	5.2.4 Only Accessibility-Supported Ways of Using Technologies	C
	Tweeter	2	5.2.4 Only Accessibility-Supported Ways of Using Technologies	C
	Internal links	3	2.4.9 Link Purpose (Link Only)	O
	Images	2	1.4.5 Images of Text	P
India	Meta tags	2	4.1.1 Parsing	R
	Headings	3	2.4.6 Headings and Labels	O
	Analytics	1	3.3.1 Error Identification	U
			3.3.2 Labels or Instructions	U
			3.3.3 Error Suggestion	U
			3.3.4 Error Prevention (Legal, Financial, Data)	U
	URL format	3	4.1.2 Name, Role, Value	R
	Internal Links	4	2.4.9 Link Purpose (Link Only)	O
	Popularity	2	3.2.4 Consistent Identification	U
	Printability	2	5.2.4 Only Accessibility-Supported Ways of Using Technologies	C
	Mobile	3	5.2.4 Only Accessibility-Supported Ways of Using Technologies	C
	Amount of content	2	3.1. Readable	U
	Server Behaviour	2	3.2.5 Change on Request	U
	Images	2	1.4.5 Images of Text	P
Philippines	Meta tags	2	4.1.1 Parsing	R
	Headings	3	2.4.6 Headings and Labels	O
	Amount of content	2	3.1. Readable	U
	Server behaviour	2	3.2.5 Change on Request	U
	Images	2	1.4.5 Images of Text	P
	Popularity	2	3.2.4 Consistent Identification	U
	Internal links	4	2.4.9 Link Purpose (Link Only)	O
Jordan	Tweeter	2	5.2.4 Only Accessibility-Supported Ways of Using Technologies	C
	Internal links	4	2.4.9 Link Purpose (Link Only)	O
	Headings	3	2.4.6 Headings and Labels	O
	Server behaviour	2	3.2.5 Change on Request	U
	Amount of content	2	3.1. Readable	U
	Images	2	1.4.5 Images of Text	P
UAE	Tweeter	2	5.2.4 Only Accessibility-Supported Ways of Using Technologies	C
	Meta Tags	2	4.1.1 Parsing	R
	Server behaviour	2	3.2.5 Change on Request	U
	Heading	3	2.4.6 Headings and Labels	O
	Popularity	2	3.2.4 Consistent Identification	U
	Amount of content	2	3.1. Readable	U
	Internal links	4	2.4.9 Link Purpose (Link Only)	O
Kyrgyzstan	Tweeter	2	5.2.4 Only Accessibility-Supported Ways of Using Technologies	C
	Analytics	1	3.3.1 Error Identification	U
			3.3.2 Labels or Instructions	U
			3.3.3 Error Suggestion	U
			3.3.4 Error Prevention (Legal, Financial, Data)	U
	Meta tags	2	4.1.1 Parsing	R
	Headings	3	2.4.6 Headings and Labels	O
	Internal links	4	2.4.9 Link Purpose (Link Only)	O
	Server behaviour	2	3.2.5 Change on Request	U
	Popularity	2	3.2.4 Consistent Identification	U
	Amount of content	2	3.1. Readable	U
	URL format	3	4.1.2 Name, Role, Value	R
	Images	2	1.4.5 Images of Text	P
Maldives	Tweeter	2	5.2.4 Only Accessibility-Supported Ways of Using Technologies	C
	Printability	2	5.2.4 Only Accessibility-Supported Ways of Using Technologies	C
	Meta tags	2	4.1.1 Parsing	R
	Internal links	4	2.4.9 Link Purpose (Link Only)	O
	URL format	3	4.1.2 Name, Role, Value	R
	Images	2	1.4.5 Images of Text	P
	Amount of content	2	3.1. Readable	U
	Popularity	2	3.2.4 Consistent Identification	U
Hong Kong	Tweeter	2	5.2.4 Only Accessibility-Supported Ways of Using Technologies	C
	Heading	3	2.4.6 Headings and Labels	O
	Amount of content	2	3. Understandable	U
	Server behaviour	2	3.2.5 Change on Request	U
	Popularity	2	3.2.4 Consistent Identification	U
Qatar	Printability	2	5.2.4 Only Accessibility-Supported Ways of Using Technologies	C
	Meta tags	2	4.1.1 Parsing	R
	Amount of content	2	3.1. Readable	U
	Images	2	1.4.5 Images of Text	O
	Popularity	2	3.2.4 Consistent Identification	U
	Internal links	4	2.4.9 Link Purpose (Link Only)	O
	Heading	3	2.4.6 Headings and Labels	O
	Tweeter	2	5.2.4 Only Accessibility-Supported Ways of Using Technologies	C

**Table A4:** Accessibility issues in the website of each country by WAVE.

Website Region	WAVE			
	Violations	No. of Occurrence	Successive Criteria	Principles
Estonia	-	-	-	-
France	Empty button	3	Non-text Content (Level A)	P
Greece	Empty Link	4	2.4.4 Link Purpose (In Context) (Level A)	O
Ireland-1	Empty button	1	1.1.1 Non-text Content (Level A)	P
	Missing alternative Text	1	1.1.1 Non-text Content (Level A)	P
	Empty Link	1	2.4.4 Link Purpose (In Context) (Level A)	O
Ireland-2	Empty button	2	1.1.1 Non-text Content (Level A)	P
	Missing alternative Text	3	1.1.1 Non-text Content (Level A)	P
	Empty Link	1	2.4.4 Link Purpose (In Context) (Level A)	O
Latvia	-	-	-	-
Luxembourg	Broken ARIA references	2	3.2.1 On Focus	U
Netherland	Broken ARIA references	4	3.2.1 On Focus	U
Iceland	Linked Image missing	1	2.4.9 Link Purpose (Link Only)	O
Switzerland	Empty button	3	1.1.1 Non-text Content (Level A)	P
	Empty Link	1	2.4.4 Link Purpose (In Context) (Level A)	O
Croatia	Broken skip link	4	2.4.9 Link Purpose (Link Only)	O
Germany	Linked image missing alternative text	3	2.4.4 Link Purpose (In Context) (Level A)	O
	Broken ARIA references	1	3.2.1 On Focus	U
	Empty button	5	1.1.1 Non-text Content (Level A)	P
	Empty heading	3	2.4.6 Headings and Labels	O
	Missing form Label	3	3.3.2 Labels or Instructions (Level A)	U
	Language missing	1	3.1.2 Language of parts (level AA)	U
	Empty Link	15	2.4.4 Link Purpose (In Context) (Level A)	O
Austria	Language missing	5	3.1.2 Language of parts (level AA)	U
	Empty Link	1	2.4.4 Link Purpose (In Context) (Level A)	O
India	Missing alternative Text	1	1.1.1 Non-text Content (Level A)	P
	Linked Image missing	2	2.4.9 Link Purpose (Link Only)	O
	Missing form Label	1	3.3.2 Labels or Instructions (Level A)	U
	Language missing	1	3.3.2 Labels or Instructions (Level A)	U
	Empty Link	3	2.4.4 Link Purpose (In Context) (Level A)	O
	Broken skip link	1	2.4.9 Link Purpose (Link Only)	O
	Missing alternative Text	4	1.1.1 Non-text Content (Level A)	P
	Linked Image missing	16	2.4.9 Link Purpose (Link Only)	O
Philippines	Empty Link	2	2.4.4 Link Purpose (In Context) (Level A)	O
	Empty heading	2	2.4.6 Headings and Labels	O
	Linked Image missing	12	2.4.9 Link Purpose (Link Only)	O
	Language missing	1	3.1.2 Language of parts (level AA)	U
	Empty Link	1	2.4.4 Link Purpose (In Context) (Level A)	O
	Missing alternative Text	3	1.1.1 Non-text Content (Level A)	P
	Linked Image missing	4	2.4.9 Link Purpose (Link Only)	O
Jordan	Missing form Label	1	3.3.2 Labels or Instructions (Level A)	U
	Linked Image missing	9	2.4.9 Link Purpose (Link Only)	O
	Missing form Label	1	3.3.2 Labels or Instructions (Level A)	U
	Empty Link	1	2.4.4 Link Purpose (In Context) (Level A)	O
	Linked Image missing	35	2.4.9 Link Purpose (Link Only)	O
	Empty Link	12	2.4.4 Link Purpose (In Context) (Level A)	O
UAE	Empty heading	1	2.4.6 Headings and Labels	O
	Empty Link	1	2.4.4 Link Purpose (In Context) (Level A)	O
	Missing alternative Text	3	1.1.1 Non-text Content (Level A)	P
	Linked Image missing	4	2.4.9 Link Purpose (Link Only)	O
	Missing form Label	1	3.3.2 Labels or Instructions (Level A)	U
Kyrgyzstan	Linked Image missing	9	2.4.9 Link Purpose (Link Only)	O
	Missing form Label	1	3.3.2 Labels or Instructions (Level A)	U
	Empty Link	1	2.4.4 Link Purpose (In Context) (Level A)	O
Maldives	Linked Image missing	35	2.4.9 Link Purpose (Link Only)	O
	Empty Link	12	2.4.4 Link Purpose (In Context) (Level A)	O
Hong Kong	-	-	-	-
Qatar	Empty heading	1	2.4.6 Headings and Labels	O
	Empty Link	1	2.4.4 Link Purpose (In Context) (Level A)	O

**Table A5:** Accessibility issues in the website of each country by WEB accessibility.

Website Region	WEB Accessibility			
	Violations	No. of Occurrence	Successive Criteria	Principles
Estonia	Provide alternative text for images	15	1.1 Text Alternatives	P
	Ensure ARIA regions, landmarks and HTML sections are identifiable	1	3.2.1 On Focus	U
	Ensure sub-lists are marked up properly	1	3.3.2 Labels or Instructions	U
France	-	-	-	-
Greece	Ensure link text is meaningful within context	4	2.4.4 Link Purpose (In Context)	O
	Ensure sub-lists are marked up properly	2	3.3.2 Labels or Instructions	U
Ireland-1	Provide alternative text for images	2	1.1 Text Alternatives	P
	Ensure link text is meaningful within context	3	2.4.4 Link Purpose (In Context)	O
	Provide a descriptive dialog title	1	3.3.2 Labels or Instructions	U
Ireland-2	Provide alternative text for images	2	1.1 Text Alternatives	P
	Ensure link text is meaningful within context	3	2.4.4 Link Purpose (In Context)	O
	Provide a descriptive dialog title	1	3.3.2 Labels or Instructions	U
Latvia	Ensure ARIA regions, landmarks and HTML sections are identifiable	1	3.2.1 On Focus	U
Luxembourg	Provide alternative text for images	4	1.1 Text Alternatives	P
	Ensure ARIA roles, states, and properties are valid	1	3.2.1 On Focus	U
Netherland	Provide alternative text for images	103	1.1 Text Alternatives	P
	Ensure ARIA roles, states, and properties are valid	1	3.2.1 On Focus	U
Iceland	Ensure link text is meaningful within context	1	2.4.4 Link Purpose (In Context)	O
	Ensure ARIA roles, states, and properties are valid	1	3.2.1 On Focus	U
	Ensure pages use the title element	1	2.4.2 Page Titled	O
Switzerland	Ensure link text is meaningful within context	5	2.4.4 Link Purpose (In Context)	O
	Ensure ARIA regions, landmarks and HTML sections are identifiable	4	3.2.1 On Focus	U
Croatia	Provide alternative text for images	12	1.1 Text Alternatives	P
	Ensure markup documents contain well-formed elements	5	3.3.2 Labels or Instructions	U
	Ensure images provide informative alternative text	1	1.4.5 Images of Text	P
Germany	Ensure the language of a document is set	1	3.1.2 Language of Parts	U
Austria	-	-	-	-
India	Provide alternative text for images	3	1.1 Text Alternatives	P
	Ensure the language of a document is set	1	3.1.2 Language of Parts	U
	Ensure the marquee element is avoided	1	3.2.1 On Focus	U
	Ensure embedded elements and canvas elements provide a meaningful text equivalent	1	3.1.5 Reading Level	U
	Ensure link text is meaningful within context	1	2.4.4 Link Purpose (In Context)	O
	Avoid use of placeholder values to label or explain input	1	3.3.2 Labels or Instructions	U
Philippines	Provide alternative text for images	4	1.1 Text Alternatives	P
	Avoid use of placeholder values to label or explain input	18	3.3.2 Labels or Instructions	U
	Ensure data table headers are properly identified	1	2.4.6 Headings and Labels	O
	Ensure frame titles are meaningful	3	2.4.10 Section Headings	O
Jordan	Provide alternative text for images	8	1.1 Text Alternatives	P
	Ensure the language of a document is set	1	3.1.2 Language of Parts	U
	Ensure link text is meaningful within context	12	2.4.4 Link Purpose (In Context)	O
	Ensure frame titles are meaningful	1	2.4.10 Section Heading	O
	Ensure containing elements allow text resize without loss of functionality.	1	1.4.4 Resize text	P
UAE	Provide alternative text for images	7	1.1 Text Alternatives	P
	Avoid use of placeholder values to label or explain input	1	3.3.2 Labels or Instructions	U
	Ensure containing elements allow text resize without loss of functionality.	1	1.4.4 Resize text	P
	Provide a valid label for form fields	2	2.5.3 Label in Name	O
Kyrgyzstan	Provide alternative text for images	8	1.1 Text Alternatives	P
	Ensure link text is meaningful within context	11	2.4.4 Link Purpose (In Context)	O
	Avoid use of placeholder values to label or explain input	1	3.3.2 Labels or Instructions	U
	Ensure ARIA regions, landmarks and HTML sections are identifiable	4	3.2.1 On Focus	U
Maldives	Provide alternative text for images	1	1.1 Text Alternatives	P
	Ensure link text is meaningful within context	12	2.4.4 Link Purpose (In Context)	O
	Ensure data table headers are properly identified	12	2.4.10 Section Heading	O
	Ensure frame titles are meaningful	2	2.4.10 Section Headings	O
	Ensure containing elements allow text resize without loss of functionality.	1	1.4.4 Resize text	P
	Ensure links do not directly target images	35	2.4.9 Link Purpose (Link Only)	O
	Ensure list items are found in a list container	2	3.2.1 On Focus	U
	Ensure ARIA roles, states, and properties are valid	1	3.2.1 On Focus	U
Hong Kong	Provide alternative text for images	1	1.1 Text Alternatives	P
	Ensure link text is meaningful within context	1	2.4.4 Link Purpose (In Context)	O
Qatar	Provide alternative text for images	1	1.1 Text Alternatives	P
